# Global Prevalence Estimates of *Toxascaris leonina* Infection in Dogs and Cats

**DOI:** 10.3390/pathogens9060503

**Published:** 2020-06-23

**Authors:** Ali Rostami, Seyed Mohammad Riahi, Vahid Fallah Omrani, Tao Wang, Andreas Hofmann, Aliyar Mirzapour, Masoud Foroutan, Yadolah Fakhri, Calum N. L. Macpherson, Robin B. Gasser

**Affiliations:** 1 Infectious Diseases and Tropical Medicine Research Center, Health Research Institute, Babol University of Medical Sciences, Babol, Iran; 2 Department of Epidemiology and Biostatistics, Faculty of Health, Cardiovascular Diseases Research Center, Birjand University of Medical Sciences, Birjand, Iran; riahim61@gmail.com; 3 Cellular and Molecular Biology Research Center, Shahid Beheshti University of Medical Sciences, Tehran, Iran; v.omrani@gmail.com; 4 Department of Veterinary Biosciences, Melbourne Veterinary School, Faculty of Veterinary and Agricultural Sciences, University of Melbourne, Parkville, VIC, Australia; tao.wang1@unimelb.edu.au (T.W.); a.hofmann@structuralchemistry.org (A.H.); 5 Innovative Medical Research Center, Department of Medical Parasitology and Mycology, School of Medicine, Mashhad Branch, Islamic Azad University, Mashhad, Iran; alimirzapour20@yahoo.com; 6 Department of Parasitology, Abadan Faculty of Medical Sciences, Abadan, Iran; masoud_foroutan_rad@yahoo.com; 7 Food Health Research Center, Department of Environmental Health Engineering, Hormozgan University of Medical Sciences, Bandar Abbas, Iran; ya.fakhri@gmail.com; 8 School of Graduate Studies, School of Veterinary Medicine, St. George’s University, WINDREF, Grenada, West Indies; CMacpherson@sgu.edu

**Keywords:** *Toxascaris leonina*, global prevalence, dogs, cats, meta-analysis

## Abstract

*Toxascaris leonina* is an ascaridoid nematode of dogs and cats; this parasite affects the health of these animals. This study estimated the global prevalence of *Ta. leonina* infection in dogs and cats using random effects meta-analysis as well as subgroup, meta-regression and heterogeneity analyses. The data were stratified according to geographical region, the type of dogs and cats and environmental variables. A quantitative analysis of 135 published studies, involving 119,317 dogs and 25,364 cats, estimated prevalence rates of *Ta. leonina* in dogs and cats at 2.9% and 3.4%, respectively. Prevalence was highest in the Eastern Mediterranean region (7.2% for dogs and 10.0% for cats) and was significantly higher in stray dogs (7.0% vs. 1.5%) and stray cats (7.5% vs. 1.8%) than in pets. The findings indicate that, worldwide, ~26 million dogs and ~23 million cats are infected with *Ta. leonina*; these animals would shed substantial numbers of *Ta. leonina* eggs into the environment each year and might represent reservoirs of infection to other accidental or paratenic hosts. It is important that populations of dogs and cats as well as other canids and felids be monitored and dewormed for *Ta. leonina* and (other) zoonotic helminths.

## 1. Introduction

Archeological findings dating back 320 centuries provide evidence that humans and animals (including dogs and cats) co-habited and benefited from their association through mutual protection, hunting, shepherding and transport [1,2]. There are numerous breeds of dogs and cats, which have a wide diversity of roles and are most commonly kept as companions [3,4]. The human-animal bond provides benefits with regard to mental health and physical well-being [1]. Working dogs are engaged as assistants to entertain, shepherd livestock, conduct search and rescue and detect illicit food, drugs and human trafficking [4,5,6,7]. Dogs and cats play an important role in comparative medical studies of diabetes, narcolepsy and cancers [4,8,9,10]. Dogs are increasingly being used for the early detection of cancers [11], and it has been hypothesized that some canine infections may reduce the severity of human infection, such as the current pandemic with COVID-19 [12].

Global dog and cat populations are ubiquitous and are estimated at 900 million (500 million of which are stray or feral) and 700 million (480 million of which are stray), respectively (https://www.worldatlas.com, https://www.carodog.eu and https://www.carocat.eu). These figures are likely underestimated because dogs and cats are not registered in many countries and roam freely. In 2019, it was estimated that about one quarter of all European households owned at least one pet; in 2018, the total numbers of companion dogs and cats were estimated at 85.2 and 103 million, respectively (https://www.statista.com). In the USA, ~63.4 and 42.7 million families owned a dog or a cat (https://www.iii.org). Total numbers of dogs owned in the USA are estimated at 89.7 million and cats at 94.2 million (https://www.iii.org). In Australia, 40% of families have dogs, 27% have cats and 61% have either or both; in 2019, totals of 5.1 million pet dogs and 3.8 million pet cats were estimated (https://animalmedicinesaustralia.org.au). The close association of domestic and stray dogs and cats with humans necessitates attention to their health and welfare, as they can pose a public health threat through more than 60 zoonotic infections as well as scratches, bites, allergies, accidents and noise pollution [6,13,14,15]. 

Parasitic roundworms (nematodes) of the family Ascarididae (“ascarids”) cause amongst the most widespread and important zoonotic infections [16,17,18]. Ascarids of canids and felids are relatively large nematodes which, as dioecious adult worms, usually inhabit the lumen of the small intestines (rarely stomach or large intestine), where they live on gut contents. Adult worms of *Toxocara canis* and *Toxocara cati* are commonly found in dogs and cats; *Toxocara malaysiensis* is found in cats in parts of Asia [19,20,21,22], and *Toxascaris leonina* infects both dogs and cats [23]. Both *T. canis* and *T. cati* can cause serious disease in puppies and kittens, whereas *Ta. leonina* is considered less pathogenic. 

The life cycles of *Ta. leonina, T. canis* and *T. cati* are well known, but that of *T. malaysiensis* has yet to be described [3,24,25,26,27,28,29,30,31]. While *Ta. leonina* is directly transmitted via the oral route, *T. canis* and *T. cati* both have oral and/or transmammary transmission, and *T. canis* is also transmitted transplacentally. These routes are, to some extent, governed by the age, sex and immune status of the animals. Disease (toxocariasis) in dogs caused by *T. canis* manifests as unthriftiness and pot-bellied appearance, with intermittent diarrhoea and, in some cases, palpably thickened small intestine. Vomiting, sneezing and coughing can occur in experimental infection. *T. cati* infection in kittens is similar to that described for less severe *T. canis* infection in puppies. *Ta. leonina* is less pathogenic in young animals, undertaking (as a larval stage) only limited migration into the intestinal wall, and has a longer prepatent period (48–72 days) than *T. canis* (20–35 days) and *T. cati* (25–42 days)—these characteristics of *Ta. leonina* allow puppies and kittens to grow and develop prior to the health impacts of the adult worms [23]. 

*T. canis* and *T. cati* can infect paratenic or accidental host animals, including rodents, lagomorphs, birds [23,25,31,32,33,34,35] and also humans [36]. *Ta. leonina* is only transmitted orally, and the larvae have been recorded to infect paratenic hosts, such as mice, rabbits and chickens and occasionally humans as accidental hosts [23,25,31,32,33,34,35]. This species has been occasionally implicated in human infection and disease [37,38,39], but its zoonotic potential has been questioned [14,40]. 

Although rarely reported, it is possible that transmission to humans may be more common than presently recognized, particularly in geographical regions in which *Ta. leonina* infection in canids and felids is endemic and prevalent [38,40]. Epidemiological studies of *Ta. leonina* are scattered and have often been small/limited. Investigations have been conducted in selected geographical regions, but there has been no comprehensive review of the literature or attempt to estimate the prevalence of *Ta. leonina* infection in dogs or cats worldwide. Recently, several reviews were published on *T. canis* and *T. cati* [16,17,18]. The present study is the first comprehensive review and meta-analysis to estimate the pooled global prevalence of *Ta. leonina* infection and associated risk factors in dogs and cats.

## 2. Results

### 2.1. Eligible Studies, Their Characteristics and Data Sets

**Figure 1:**
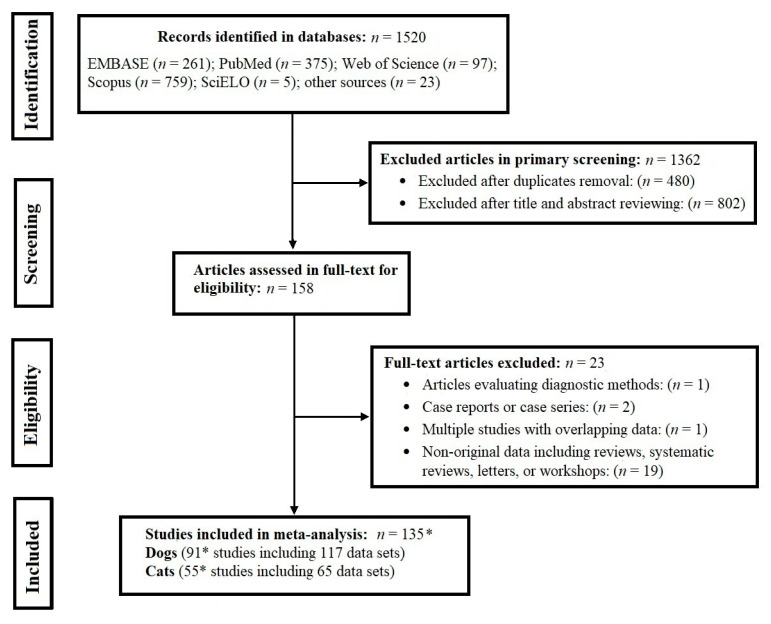
Flow diagram showing the search and selection methodology used, which follows PRISMA guidelines. Some studies investigated both dogs and cats (*).

Figure 1 summarises the numbers of publications at each stage of the process. Our search resulted in the identification of 1520 articles, 1362 of which were excluded, following the removal of duplicates and the screening of titles and abstracts. In total, 158 articles with full-texts were assessed for eligibility; 91 and 55 studies of dogs and cats, containing 117 and 65 data sets, respectively, were included in this meta-analysis. These studies provided data for 119,317 dogs and 25,364 cats from 40 and 28 different countries, respectively, from all continents. In total, 74,794/15,114 animals (i.e. dogs/cats) were examined in Europe, 30,880/4222 in North America, 5736/2784 in the Western Pacific region, 3409/1877 in the Eastern Mediterranean region, 2577/319 in Africa, 345/1048 in South America and 1576/0 in South-East Asia. In total, 96,187 and 19,200 pet dogs and cats, 10,031 and 4169 stray dogs and cats and 5966 and 1995 indeterminate (no specified type) of dogs and cats were studied, respectively. Moreover, 7133 working dogs were also tested for *Ta. leonina* infection. The salient descriptive characteristics of these studies are given in Tables S1 and S2.

### 2.2. Global and Regional Prevalence Rates of Toxascaris leonina Infection in Dogs

**Figure 2:**
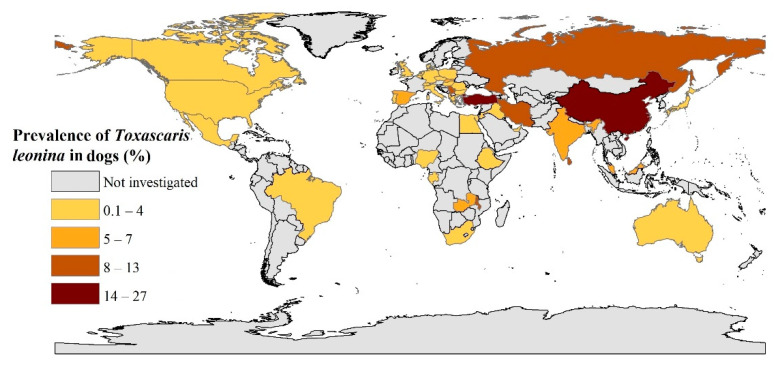
Prevalence of *Toxascaris leonina* infection in dogs in different countries using geographic information system (GIS).

For the 117 data sets, 3229 of 119,317 dogs were diagnosed as having *Ta. leonina* infection, resulting in an overall, pooled global prevalence of 2.9% (95% CI, 2.2–3.8) (Table 1; Figure 2), with evidence of heterogeneity among studies (*I*^2^ = 98.0%, *P* < 0.001). In WHO-regions, the pooled prevalences (in descending order, with the range) were 7.2% (3.5–12.0%) in the Eastern Mediterranean region, 5.7% (1.4–12.2%) in South-East Asia, 3.6% (1.2–6.9%) in Africa, 2.6% (1.6–3.8%) in Europe, 2.0% (1.1–3.2%) in North America), 1.0% (0.1–3.4%) in the Western Pacific and 0.6% (0.1–2.1%) in South America. For countries with three or more eligible data sets, Iran (10.8%), India and Spain (5.3%), Slovakia (5.0%) and Canada (3.6%) exhibited some of the highest prevalences. Additional details pertaining to the prevalence of *Ta. leonina* infection in dogs in WHO-regions and individual countries are given in Table 1 and Figure 2.

### 2.3. Global and Regional Prevalence Rates of Toxascaris leonina Infection in Cats 

**Figure 3:**
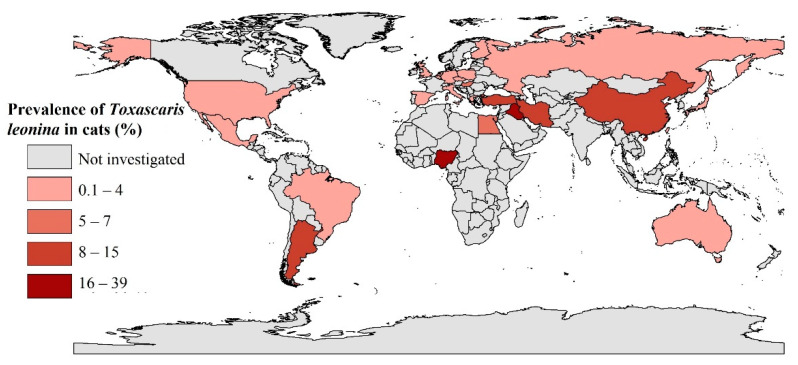
Prevalence of *Toxascaris leonina* infection in cats in different countries using geographic information system (GIS).

For the 65 data sets, 511 of 25,364 cats were diagnosed as having *Ta. leonina* infection, resulting in an overall pooled global prevalence of *Ta. leonina* infection in cats of 3.4% (95% CI, 2.3–4.8%; Table 2), with evidence of heterogeneity among studies (*I*^2^ = 95.5%, *P* < 0.001). In WHO-regions, pooled prevalences were 10.0% (3.3–19.4%) in the Eastern Mediterranean region, 4.3% (0.3–11.9%) in South America, 1.9% (0.9–3.3%) in Europe, 1.4% (0.4–2.8%) in the Western Pacific and 0.01% (0.0–0.1%) in North America. For Africa, we identified only three eligible data sets from two publications for Nigeria, from which a prevalence of 38.7% for *Ta. leonina* infection was calculated. There were no data for the South East Asian region. For countries with three or more eligible data sets, the highest prevalences were inferred for Nigeria (38.7%), Iran (13.7%), Russia (4.0%) and Brazil (3.3%). Other details pertaining to the prevalence of *Ta. leonina* infection in cats in WHO-regions and individual countries are given in Table 2 and Figure 3.

### 2.4. Prevalence According to Type of Animals and Selected Study Characteristics 

Subgroup analyses conducted according to the “type of animal” studied (Table 3) revealed that the prevalence of *Ta. leonina* infection in stray dogs (7.0%, 4.3–10.3%) was significantly higher (*P* < 0.001) than in working (3.9%, 1.9–7.2%), “indeterminate-type” (3.0%, 0.8–6.5%) or pet (1.5%, 0.9–2.3%) dogs (*P* < 0.001). Moreover, the global prevalence of *Ta. leonina* infection was 7.5% (4.0–11.8%) in stray, 3.3% (2.2–4.6%) in indeterminate-type and 1.8% (0.9–2.9%) in pet cats, with a significant difference between these subgroups (*P* < 0.001). Subgroup analyses, conducted according to sample size, revealed the lowest (1.0%) and highest (4.0%) prevalences in studies with sample sizes of ≤500 and ≥5000 animals. Studies conducted after 2005 indicated non-significantly lower prevalences (*P* = 0.09). With regard to study quality, those with a moderate risk of bias (7.5%) had significant higher prevalences than studies with a low risk of bias (2.5%) (*P* < 0.001). More detail is given in Table 3.

### 2.5. Impact of Socio-demographic, Geographical and Climatic Parameters on Prevalence 

We also performed subgroup analyses with respect to socio-demographic, geographical and climate parameters, to attempt to establish the source of heterogeneity and also the effects of these parameters on the prevalence of *Ta. leonina* infection in dogs and cats (Table 4). When the pooled prevalence was stratified according to the income-level of people in a country, the highest prevalences were estimated for countries with low to middle income-levels (7.5%, 3.8–12.2%) and the lowest for those with high income-levels (1.4%, 1.0–1.8%). According to geographical latitude, the highest prevalence was seen at latitudes of 0–10° (9.7%, 2.7–19.9%) and the lowest at latitudes of 40–50° (1.8%, 1.2–2.4%). With respect to longitude, the highest and lowest prevalences were estimated at longitudes of 40–50° (6.9%, 5.6–19.0%) and ≥ 120° (0.4%, 0.1–0.8%), respectively. The highest prevalences were estimated at a mean relative environmental humidity of 41–59% (6.9%, 4.3–9.9%), a mean environmental temperature of 19–25 °C (6.9%, 3.5–11.1%) and a precipitation range of 251–500 mm (5.4%, 3.5–7.7%). More detail is given in Table 4. 

With respect geographical parameters, meta-regression analysis showed a non-significant decreasing trend in prevalence with increasing geographical latitude (coefficient [*C*] = −0.0006, *P* = 0.14) and longitude (*C* = 0.00009, *P* value = 0.37). Considering climatic parameters, a marginally-significant decreasing trend was observed for increasing mean relative humidity (*C* = 0.001, *P* = 0.05). Moreover, a non-significant increasing trend in prevalence was seen with increasing mean environmental temperature (*C* = 0.0008; *P* = 0.32). Finally, a non-statistically significant decreasing trend was seen for increasing annual precipitation (*C* = −00002, *P* = 0.09) (Figure S1; panels A–F). 

## 3. Discussion 

Here, we undertook a systematic review and meta-analysis of published studies to estimate the prevalence and distribution of the *Ta. leonina* infection in dogs and cats worldwide. The global prevalence of *Ta. leonina* infection in dogs was 2.9% (2.2–3.7%) and 3.3% (2.2–4.6%) in cats. Worldwide, we estimated that ~26 million dogs and ~23 million cats are infected with *Ta. leonina*. There were significant differences in prevalence, depending on geographical region, owners’ income-levels in particular countries, type of animal (e.g., stray or pet) and study characteristics (cf. Table 1; Table 2).

The high prevalences of *Ta. leonina* infection estimated for the Eastern Mediterranean and African regions and low prevalences for the European, North American and the Western Pacific regions are in accordance with recent estimates for *T. canis* and *T. cati* infections in dogs and cats. [16,17]. These findings need to be interpreted with some caution due to the differences in the “types” and numbers of animals included in the different publications and the limited number of studies for some geographical regions (e.g., Eastern Mediterranean, Africa, South-East Asia and South America) (Tables S1 and S2). Subgroup analyses showed that stray animals and studies with low sample sizes had significant higher prevalences of *Ta. leonina* infection compared with pet animals and studies with large sample sizes, consistent with previous studies of *Toxocara* [16,17]. Subgroup analysis indicated that prevalence of *Ta. leonina* infection is significantly lower in countries with a high level of income per capita (e.g., European, Western Pacific and North American regions) compared with those with low or middle income-levels (e.g., Mediterranean, Africa and South America), again in accord with recent studies of *Toxocara* [16,17]. The latter difference might be explained by the adverse impact of socioeconomic (income- and education-levels) and political factors (including political instability or war) in some countries on veterinary care and programs to control stray animal populations.

The higher prevalences of *Ta. leonina* infection in stray dogs (6.6% *vs.* 1.5%) and cats (8.0% vs. 1.6%) compared with pets suggests a greater role of strays in contaminating the environment and facilitating transmission. The higher prevalences in stray animals was anticipated based on previous studies of *Toxocara* species [16,17] but needed to be independently evaluated, even though *Ta. leonina* belongs to the same nematode family (Ascarididae). Such animals usually/often have a poor nutritional status, are susceptible to infections, are not under veterinary care and are not treated with anti-parasitic drugs [16,41,42] and, thus, are likely “persistent” reservoirs of *Ta. leonina*. 

Subgroup and meta-regression analyses revealed that the prevalence of *Ta. leonina* infection had a non-significant decreasing trend in recent years, like *Toxocara* infections in dogs and cats [16,17]. Increased knowledge of pet owners about the importance of the health of their animals and increased anti-parasite treatments may explain, to some extent, this trend [43,44]. With regard to geographical and climatic parameters, the non-significant higher prevalence of *Ta. leonina* infection in both dogs and cats in areas with low geographical latitudes and longitudes means higher temperature, lower relative humidity and annual precipitation likely relate to beneficial survival and embryonation rates of *Ta. leonina* eggs in the environment, as suggested for *Toxocara* infections in these animals [16,17,36,45,46].

Although this systematic review is the first to explore the prevalence of *Ta. leonina* infection in dogs and cats worldwide, it has some limitations in that: (i) some “grey” literature [47]—produced by organisations external to traditional academic or commercial publishers—may have gone undetected; (ii) data were not available for numerous countries, and thus, our estimates may sometimes not be representative in all countries and regions; (iii) the main aim of most publications included was to study *T. canis* and/or *T. cati* or other small intestinal parasites, and the finding of *Ta. leonina* was a “side issue”, so precise information on sex, age and/or location of animals was often not reported; and (iv) there was significant heterogeneity among studies, which is a commonly observed feature of global prevalence studies [48,49]. The comprehensiveness of the literature search, the data from >40 countries, the large numbers of dogs and cats included and the subgroup and meta-regression analyses indicate that the prevalence estimates are relatively reliable.

## 4. Methodology

This systematic review and meta-analysis was conducted using a standard protocol, according to the Preferred Reporting Items for Systematic Reviews and Meta-Analyses (PRISMA) [50].

### 4.1. Search Strategy and Selection Criteria

Two independent investigators (A.M. and M.F.) systematically screened five international databases (i.e., Web of Science, Scopus, PubMed, EMBASE and SciELO) for peer-reviewed papers, published from 1 January 1990 to 1 July 2019, to retrieve all publicly accessible data on the prevalence of *Ta. leonina* infection in dogs and cats. No geographic or language limitations were applied to the search procedure. A combination of the following search terms was used: “*Toxascaris leonina*”, “*Ta. leonina*”, “*Toxascaris*”, “intestinal parasites”, “gastrointestinal helminth”, “endoparasites”, “epidemiology”, “prevalence”, “incidence”, “dog”, “canine”, “puppy”, “cat”, “feline”, and “kitten”, alone or combined with the Boolean operators ‘OR’ and/or ‘AND’. The online tool "Google Translate" (https://translate.google.com/) was employed to access publications in languages other than English. For the systematic review, dogs and cats were included if *Ta. leonina* eggs were detected in fecal or hair samples, or *Ta. leonina* adult worms were found upon postmortem examination. All published works retrieved were imported into the program Endnote v.X7 and duplicate records removed. Two investigators (A.R. and V.F.O.) independently screened titles and abstracts and eliminated all studies that were unequivocally assessed as irrelevant in relation to the aim of the review. The abstracts of all remaining studies were saved in separate word files for the subsequent assessment of inclusion criteria. All potentially eligible articles were downloaded from online resources; if required, additional information was obtained from corresponding authors of a particular article. 

Full texts of articles were assessed independently by two investigators (Y.F. and V.F.O.) for their suitability; any disagreement about inclusion/exclusion was resolved through discussion with the principal investigator (A.R.) to achieve a consensus. Publications were included in the current systematic review if they satisfied all of the following inclusion criteria: (1) peer-reviewed original research articles or short communications, which reported the prevalence of *Ta. leonina* in dogs or cats; (2) sample size of > 30 for dogs or cats; (3) fecal or hair examination method to detect and identify *Ta. leonina* eggs or a postmortem examination technique to identify adult *Ta. leonina*; (4) published between 1 January 1990 and 1 July 2019; (5) full-texts were available; and (6) precise information was reported on sample size(s) and the specific identity of the eggs or worms found. Publications were excluded if they did not meet all of these criteria or if they were review articles, systematic reviews, editorials or case reports.

### 4.2. Data Extraction and Quality Assessment

After assessing all eligibility criteria for each publication, relevant data and information were extracted independently by two authors (M.F. and A.M.) and collated in a Microsoft Excel spreadsheet (2016 version; Microsoft, Redmond, WA, USA) in a blinded manner. The extracted data and information were meticulously reappraised for accuracy by a third investigator (A.R.). Any disagreement or inconsistency was discussed and resolved to reach a consensus decision about inclusion or exclusion. Information from each eligible article (including the first author’s last name, publication year, study period, WHO-defined region, country, city, type of dogs and cats (pet or stray), sample size and number of *Ta. leonina*-positive samples) was extracted and entered into a spreadsheet in the program Microsoft Excel. The different types of dogs and cats studied in individual eligible published articles and reports were categorized into distinct groups (see Tables S1 and S2).

For each eligible publication, we estimated the pooled prevalences of *Ta. leonina* infection according to WHO-defined regions (Africa, Eastern Mediterranean, Europe, South-East Asia, the Americas and the Western Pacific) [51], World Bank’s income-level (https://datahelpdesk.worldbank.org), mean annual temperature, mean relative humidity, mean annual rainfall and geographical latitude and longitude; we considered North America and South America separately, because there are significant differences in terms of socio-demographic and climate conditions in these areas (https://en.wikipedia.org/wiki/Americas). Different data sources were employed to specify the geographical and climatic status of cities and regions (https://www.timeanddate.com/, https://en.climate-data.org/ and https://gps-coordinates.org/) [16]. 

To assess the quality and the risk of bias for each eligible publication, we used the Joanna Briggs Institute (JBI) Prevalence Critical Appraisal Tool [52]. Accordingly, a checklist was designed to appraise the quality of records for inclusion into this systematic review and meta-analysis (Table S3). Here, two trained authors (V.F.O. and M.F.) independently appraised the quality of each record; if a discrepancy arose, the final decision for inclusion or exclusion was made by the leader investigator (A.R.). Publications given scores of 7–10, 4–6 or 1–3 were ranked as having “low”, “moderate” and “high” risks of bias, respectively.

### 4.3. Meta-Analysis

In this systematic review, all analyses were conducted using the random effects model to estimate the pooled global prevalence of *Ta. leonina* infection in dogs and cats, as described previously [16,17,53,54]. Global and regional prevalences in WHO-regions or countries were recorded using a 95% confidence interval (CI). Heterogeneity among studies was computed using the Cochran *Q* and *I*^2^ statistics to define the degree of heterogeneity employing a cut-off value of 50% [55]. To assess the source of heterogeneity between studies, subgroup and meta-regression analyses were conducted. Subgroup analyses were carried out according to WHO-regions, types of cats and dogs, income-levels of countries, study characteristics (sample size, publication year and risk of bias), geographical latitude and longitude and climatic parameters (mean relative humidity, annual temperature and annual precipitation). In the meta-analysis, publication bias was not computed, because it is considered irrelevant for prevalence studies [56]. All statistical analyses were performed using STATA v.13 (STATA Corp., College Station, TX, USA), and a *P*-value of < 0.05 was considered as significant.

## 5. Conclusions

This study estimated overall prevalences of *Ta. leonina* infection of 2.9% (~26 million) in dogs and 3.3% (~23 million) in cats worldwide, and it intends to inform veterinary and medical practitioners about the need for intervention programs to reduce the burden of *Ta. leonina* and other ascaridoid infections in dogs and cats, particularly strays, focused on minimizing their transmission to paratenic or accidental host animals.

## Figures and Tables

**Table 1:** Global, regional and national prevalences of *Toxascaris leonina* infection in dogs, estimated from results extracted from 117 datasets from 40 countries.

WHO Region/Country	Number of Data Sets	Total Number of Samples	Number of Test-Positive Samples	Pooled Prevalence (%) Established Using Meta-Analysis (95 % CI)	Heterogeneity *I^2^* (%)
**Global**	**117**	**119,317**	**3229**	**2.9 (2.2–3.8)**	**98.0**
**Eastern Mediterranean**	**19**	**3409**	**188**	**7.2 (3.5–12.0)**	**94.6**
Iran	12	2639	167	10.8 (4.7–19.0)	96.2
Egypt	5	337	11	2.9 (0.1–10.9)	87.4
Jordan	1	340	9	2.6 (1.2–5.0)	na
Iraq	1	93	1	1.1 (0.0–5.8)	na
**South-East Asia**	**6**	**1576**	**69**	**5.7 (1.4–12.2)**	**94.0**
Sri Lanka	2	90	11	9.5 (4.0–16.7)	98.5
India	4	1486	58	5.3 (1.0–12.3)	95.4
**Africa**	**10**	**2577**	**73**	**3.6 (1.2–6.9)**	**91.5**
Nigeria	4	1418	17	1.3 (0.1–3.3)	71.9
South Africa	2	303	21	3.0 (1.3–5.4)	90.6
Gabon	1	198	1	0.5 (0.0–2.8)	na
Malawi	1	40	5	12.5 (4.2–26.8)	na
Ethiopia	1	326	9	2.8 (1.3–5.2)	na
Zambia	1	292	20	6.8 (4.2–10.4)	na
**Europe**	**55**	**74,794**	**2532**	**2.6 (1.6–3.9)**	**98.6**
Italy	7	4799	33	0.7 (0.2–1.4)	68.9
Spain	6	3595	374	5.3 (2.0–10.0)	95.2
Poland	5	4842	311	3.0 (0.9–6.2)	91.3
Germany	4	36,889	219	0.6 (0.4–0.9)	77.6
Belgium	4	3483	249	2.5 (0.0–9.2)	98.1
Greece	4	1915	70	3.2 (1.1–6.3)	87.0
Slovakia	4	1305	63	5.0 (2.9–7.6)	71.3
Portugal	3	494	3	0.3 (0.0–1.2)	0.0
Czech Republic	3	4778	46	1.0 (0.6–1.4)	31.1
Romania	2	1314	14	1.0 (0.5–1.6)	0.0
Hungary	2	490	6	0.8 (0.1–1.9)	95.7
Albania	2	713	6	0.7 (0.2–1.6)	97.7
Turkey	2	524	143	27.0 (23.2–30.9)	99.0
Russia	1	8140	970	11.9 (11.2–12.6)	na
Switzerland	1	505	7	1.4 (0.6–2.8)	na
Netherland	1	445	3	0.7 (0.1–2.0)	na
Denmark	1	178	1	0.6 (0.0–3.1)	na
England	1	171	0	0.1 (0.0–2.1)	na
Serbia	1	134	13	9.7 (5.3–16.0)	na
Bulgaria	1	80	1	1.3 (0.0–6.8)	na
**North America**	**11**	**30,880**	**204**	**2.0 (1.1–3.2)**	**96.4**
Canada	6	2647	94	3.6 (1.1–7.3)	93.9
USA	4	27,855	101	0.6 (0.2–1.1)	94.0
Mexico	1	378	9	2.4 (1.1–4.5)	na
**Western Pacific**	**15**	**5736**	**161**	**1.0 (0.1–3.4)**	**97.3**
Japan	8	3150	11	0.2 (0.0–0.6)	39.2
Australia	4	1893	3	0.1 (0.0–0.3)	12.5
China	2	616	143	19.8 (16.8–23.1)	96.9
Malaysia	1	77	4	5.2 (1.4–12.8)	0.0
**South America**	**1**	**345**	**2**	**0.6 (0.1–2.1)**	**0.0**
Brazil	1	345	2	0.6 (0.1–2.1)	0.0

**Abbreviations:** NA, not applicable; CI, confidence interval. WHO-regions (bold-type) sorted according to prevalence rates. Countries sorted according to the number of studies included.

**Table 2:** Global, regional and national prevalences of *Toxascaris leonina* infection in cats, estimated from results extracted from 65 datasets from 28 countries.

WHO Regions/Country	Number of Data Sets	Total Number of Samples	Number of Test-Positive Samples	Pooled Prevalence (%) Established Using Meta-Analysis (95 % CI)	Heterogeneity *I^2^* (%)
**Global**	**65**	**25,364**	**511**	**3.4 (2.3–4.8)**	**95.5**
**Africa**	**3**	**319**	**104**	**38.7 (20.9–58.1)**	**89.9**
Nigeria	3	319	104	38.7 (20.9–58.1)	89.9
**Eastern Mediterranean**	**10**	**1877**	**155**	**10.0 (3.3–19.4)**	**96.8**
Iran	4	316	44	13.7 (3.8–28.0)	89.5
Iraq	2	380	88	22.8 (18.7–27.2)	96.5
Egypt	2	283	20	7.0 (4.2–10.3)	95.0
Qatar	1	658	1	0.2 (0.0–0.8)	na
United Arab Emirates	1	240	2	0.8 (0.1–3.0)	na
**South America**	**5**	**1048**	**58**	**4.3 (0.3–11.9)**	**94.2**
Brazil	4	583	17	3.3 (0.0–11.6)	92.1
Argentina	1	465	41	8.8 (6.4–11.8)	na
**Europe**	**30**	**15,114**	**155**	**1.9 (0.9–3.3)**	**92.6**
Greece	5	1779	16	0.9 (0.0–3.3)	88.3
Netherland	4	1018	6	1.0 (0.0–4.4)	85.6
Spain	3	1008	15	1.3 (0.5–2.5)	47.6
Germany	3	9523	43	0.7 (0.0–5.2)	97.8
Russia	3	334	14	4.0 (2.0–6.5)	0
Italy	2	237	12	3.8 (1.6–6.7)	97.1
Turkey	2	172	17	7.8 (4.1–12.4)	73.8
England	2	142	10	1.5 (0.5–5.0)	96.5
Poland	2	90	3	0.9 (0.0–4.7)	95.2
Finland	1	411	1	0.2 (0.0–1.3)	na
Hungary	1	235	17	7.2 (4.3–11.3)	na
Czech Republic	1	135	1	0.7 (0.0–4.1)	na
Belgium	1	30	0	0.1 (0.0–11.6)	na
**Western Pacific**	**8**	**2784**	**33**	**1.4 (0.4–2.8)**	**80.2**
Australia	5	1707	27	1.6 (0.5–3.2)	75.5
Japan	1	942	2	0.2 (0.0–0.8)	na
Taiwan	1	96	1	1.0 (0.0–5.7)	na
China	1	39	3	7.7 (1.6–20.9)	na
**North America**	**9**	**4222**	**6**	**0.01 (0.0–0.1)**	**28.4**
Canada	5	976	3	0.0 (0.0–0.4)	8.0
USA	2	2888	2	0.0 (0.0–0.2)	77.0
Mexico	2	358	1	0.2 (0.0–1.1)	77.0
**South-East Asian**	**0**	**0**	**0**	**na**	**na**

**Abbreviations:** NA, not applicable; CI, confidence interval. WHO-regions (bold-type) sorted according to prevalence rates. Countries sorted according to the number of studies included.

**Table 3:** The global prevalence of *Toxascaris leonina* in dogs and cats estimated according to *a priori* defined subgroups.

Parameters/Subgroups	Number of Datasets	Total Number of Samples	Number of Test-Positive Samples	Pooled Prevalence (%) Estimated using REM (95% CI)	Heterogeneity *I^2^* (%)
**Type of dogs**					
Pet (domestic) dogs	64	96,187	1852	1.5 (0.9–2.3)	98.1
Working (domestic) dogs	16	7133	324	3.9 (1.9–7.2)	97.4
Stray (wild) dogs	28	10,031	674	7.0 (4.3–10.3)	96.7
Indeterminate (not specified type)	9	5966	379	3.0 (0.8–6.5)	97.5
**Type of cats**					
Pet (Domestic) cats	36	19,200	211	1.8 (0.9–2.9)	93.9
Stray (wild) cats	25	4169	292	7.5 (4.0–11.8)	95.7
Indeterminate (not specified type)	4	1995	8	3.3 (2.2–4.6)	83.6
**Sample size**					
≤500	140	26,003	1246	4.0 (3.0–5.1)	94.2
501–1000	21	13,909	181	1.0 (0.6–1.6)	87.7
1001–5000	14	27,408	1068	2.5 (0.9–4.9)	99.1
≥5000	7	77,361	1245	1.0 (0.1–3.0)	99.8
**Implementation year**					
1990–1995	19	16,966	374	1.9 (0.6–3.8)	96.7
1996–2000	6	5252	129	6.2 (1.8–12.8)	97.3
2001–2005	28	23,770	1005	4.7 (2.7–7.2)	98.3
2006–2010	57	68,842	689	2.3 (1.6–3.1)	96.5
2011–2015	64	25,298	1450	3.2 (2.0–4.7)	96.3
2016–2019	8	4553	93	2.4 (0.6–5.2)	95.4
**Risk of bias**					
Low risk	139	141,450	3481	2.4 (1.8–3.0)	97.9
Moderate risk	43	3231	259	7.5 (4.4–11.2)	91.6

**Abbreviations:** REM, random effect meta-analysis; CI, confidence interval.

**Table 4:** The global prevalence of *Toxascaris leonina* infection in dogs and cats estimated according to different socio-demographic and geographic parameters.

Parameter/Subgroup	Number of Data Sets	Total Number of Samples	Number of Test-Positive Samples	Pooled Prevalence (%) Established Using REM (95% CI)	Heterogeneity *I*^2^ (%)
**Income level**					
Low	30	516	15	3.2 (0.2–8.6)	79.7
Lower middle	20	4075	240	7.5 (3.8–12.2)	95.4
Upper middle	48	17,962	1684	7.4 (5.0–10.3)	97.1
High	111	122,128	1801	1.4 (1.0–1.8)	96.4
**Latitude**					
0–10°	10	2062	136	9.7 (2.7–19.9)	97.1
10–20°	9	2005	58	2.1 (0.7–4.1)	81.6
20–30°	25	6076	261	3.6 (1.3–6.8)	96.4
30–40°	52	19,708	916	4.9 (3.1–7.1)	97.4
40–50°	50	81,457	871	1.8 (1.2–2.4)	95.8
> 50°	36	33,373	1498	2.0 (0.7–3.7)	98.5
**Longitude**					
0–10°	43	48,820	1028	2.8 (1.6–4.2)	97.7
10–20°	27	24,516	240	1.6 (1.0–2.4)	97.6
20–30°	22	10,374	458	2.9 (1.4–4.8)	95.2
30–40°	10	1500	133	7.3 (2.5–14.1)	93.7
40–50°	15	3330	291	11.5 (5.6–19.0)	96.9
50–60°	14	2709	143	6.5 (2.3–12.4)	96.0
60–70°	0	0	0	na	na
70–80°	9	31,920	148	1.0 (0.5–1.8)	95.6
80–90°	3	399	24	5.6 (0.1–17.0)	86.1
90–100°	0	0	0	na	na
100–110°	12	11,816	1070	2.8 (0.5–6.6)	98.0
110–120°	7	1655	164	3.8 (0.1–14.0)	73.8
> 120°	20	7642	41	0.4 (0.1–0.8)	67.5
**Relative humidity (%)**					
< 40	7	797	96	6.4 (0.8–15.9)	94.4
41–59	30	7106	315	6.9 (4.3–9.9)	94.9
60–79	127	129,310	3166	2.4 (1.8–3.2)	98.0
≥ 80	18	7468	163	2.1 (0.7–3.9)	92.7
**Mean temperature (°C)**					
≤ 7.0	18	12,535	1139	3.5 (1.1–7.0)	97.8
7.1–13.0	68	94,938	1447	2.6 (1.9–3.4)	97.8
13.1–19.0	58	29,815	781	2.2 (1.2–3.4)	97.2
19.1–25.0	26	3815	277	6.9 (3.5–11.1)	94.9
25.1–30.0	12	3578	96	2.7 (0.7–5.8)	94.1
**Precipitation (mm)**					
0–250	18	3992	184	4.1 (1.6–7.6)	94.9
251–500	40	14,289	565	5.4 (3.5–7.7)	96.1
501–1000	80	74,762	2540	2.7 (1.8–3.7)	97.9
1001–2000	40	51,130	444	1.8 (1.1–2.6)	96.6
> 2000	4	508	6	1.0 (0.1–3.4)	68.2

**Abbreviations:** REM, random effect meta-analysis; CI, confidence interval.

## Supplementary Materials

The following are available online at https://www.mdpi.com/2076-0817/9/6/503/s1. Figure S1: Results of meta-regression analyses of the prevalence of *Toxascaris leonina* infection in dogs and cats according to: (panel A) demonstrating a statistically non-significant decreasing trend in prevalence over time in more recent years; (panels B and C) geographical latitude and longitude, showing statistically non-significant downward trend in prevalence with increasing geographical latitude and longitude; (panel D) relative humidity, showing statistically significant downward trend in prevalence with increasing relative humidity; and (panels E and F) mean environmental temperature and annual precipitation, showing non-statistically significant upward and downward trends in prevalence in areas with a higher mean temperature and relative humidity, respectively. “ES” refers to effect size (= prevalence rates). Table S1: Main characteristics of all eligible studies reporting prevalence of *Toxascaris leonina* infection in dogs. Table S2: Main characteristics of all eligible studies reporting prevalence of *Toxascaris leonina* infection in cats. Table S3: Questions from the Joanna Briggs Institute Prevalence Critical Appraisal Tool.
