# Supplementary material for: Global Prevalence Estimates of Toxascaris leonina Infection in Dogs and Cats
Source: Pathogens. 2020 Jun 23;9(6):503. doi: 10.3390/pathogens9060503 (PMC7350385; doi:10.3390/pathogens9060503)
Supplement: Supplementary file 1 [file pathogens-09-00503-s001.pdf]

Table 1. Main characteristics of all eligible studies reporting prevalence of *Toxascaris leonina* infection in dogs.

WHO-region/reference	Reference	Type of dog (pet or stray)	Country	Income-level	Sample size	Number of test-positive samples for *Ta. leonina*	Risk of bias (quality assessment)
**Africa**							
Minnaar et al. (2002)	[1]	Stray (wild) dogs	South Africa	Upper middle	63	20	Moderate Risk
Davoust et al. (2008)	[2]	Stray (wild) dogs	Gabon	Upper middle	198	1	Low Risk
Sowemimo (2007)	[3]	Pet (Domestic) dogs	Nigeria	Lower middle	269	9	Low Risk
Sowemimo and Asaolu (2008)	[4]	Pet (Domestic) dogs	Nigeria	Lower middle	959	6	Low Risk
Mukaratirwa and Singh (2010)	[5]	Stray (wild) dogs	South Africa	Upper middle	240	1	Low Risk
Awoke et al. (2011)	[6]	Indeterminate (not specified) type	Ethiopia	Low	326	9	Low Risk
Bwalya et al. (2011)	[7]	Pet (Domestic) dogs	Zambia	Lower middle	292	20	Low Risk
Mahmuda et al. (2012)	[8]	Working (domestic) dogs	Nigeria	Lower middle	40	1	Moderate Risk
Edosomwan et al. (2012)	[9]	Pet (Domestic) dogs	Nigeria	Lower middle	150	1	Low Risk
Alvåsen et al. (2016)	[10]	Pet (Domestic) dogs	Malawi	Low	40	5	Moderate Risk
**Eastern Mediterranean**							
El-Shehabi et al. (1999)	[11]	Stray (wild) dogs	Jordan	Upper middle	340	9	Low Risk
Dalimi et al. (2006)	[12]	Stray (wild) dogs	Iran	Upper middle	83	27	Low Risk
Sabry and Hossein (2009)	[13]	Stray (wild) dogs	Egypt	Lower middle	27	9	Moderate Risk
Zare-Bidaki et al. (2010)	[14]	Working (domestic) dogs	Iran	Upper middle	59	9	Moderate Risk
Mirzaei and Fooladi (2012)	[15]	Pet (Domestic) dogs	Iran	Upper middle	70	1	Low Risk
Mirzaei and Fooladi (2013)	[16]	Pet (Domestic) dogs	Iran	Upper middle	100	1	Low Risk
Beiromvand et al. (2013)	[17]	Pet (Domestic) dogs	Iran	Upper middle	28	11	Moderate Risk
Beiromvand et al. (2013)	[17]	Stray (wild) dogs	Iran	Upper middle	49	11	Moderate Risk
Adinezadeh et al. (2013)	[18]	Stray (wild) dogs	Iran	Upper middle	100	53	Low Risk
Ahmed et al. (2014)	[19]	Working (domestic) dogs	Egypt	Lower middle	120	0	Moderate Risk
Ahmed et al. (2014)	[19]	Pet (Domestic) dogs	Egypt	Lower middle	60	1	Moderate Risk
Awadallah et al. (2015)	[20]	Working (domestic) dogs	Egypt	Lower middle	70	0	Low Risk
Awadallah et al. (2015)	[20]	Pet (Domestic) dogs	Egypt	Lower middle	60	1	Low Risk
Sardarian et al. (2015)	[21]	Pet (Domestic) dogs	Iran	Upper middle	243	7	Low Risk
Emamapour et al. (2015)	[22]	Stray (wild) dogs	Iran	Upper middle	100	7	Moderate Risk
Sardarian et al. (2015)	[21]	Stray (wild) dogs	Iran	Upper middle	1257	32	Low Risk
Sarvi et al. (2016)	[23]	Working (domestic) dogs	Iran	Upper middle	100	4	Low Risk
Kohansal et al. (2017)	[24]	Indeterminate (not specified) type	Iran	Upper middle	450	4	Moderate Risk
Al-Jassim et al. (2017)	[25]	Pet (Domestic) dogs	Iraq	Upper middle	93	1	Moderate Risk
**Europe**							
Vanparijs et al. (1991)	[26]	Stray (wild) dogs	Belgium	High	2324	236	Low Risk
Epe et al. (1993)	[27]	Working (domestic) dogs	Germany	High	3329	37	Low Risk
Svobodova et al. (1995)	[28]	Pet (Domestic) dogs	Czech Republic	High	458	3	Low Risk
Causape et al. (1996)	[29]	Pet (Domestic) dogs	Spain	High	81	2	Moderate Risk
Overgaauw and Boersema (1998)	[30]	Pet (Domestic) dogs	Netherlands	High	445	3	Low Risk
Masnik (2000)	[31]	Stray (wild) dogs	Poland	High	110	1	Low Risk
Fok et al. (2001)	[32]	Pet (Domestic) dogs	Hungary	High	427	3	Low Risk
Fok et al. (2001)	[32]	Pet (Domestic) dogs	Hungary	High	63	3	
Barutzki et al. (2003)	[33]	Pet (Domestic) dogs	Germany	High	8438	42	Low Risk
Benito et al. (2003)	[34]	Stray (wild) dogs	Spain	High	1040	81	Low Risk
Borecka et al. (2005)	[35]	Pet (Domestic) dogs	Poland	High	3774	284	Low Risk

Sager et al. (2006)	[36]	Pet (Domestic) dogs	Switzerland	High	505	7	Low Risk
Senlik et al. (2006)	[37]	Working (domestic) dogs	Turkey	Upper middle	352	77	Low Risk
Dubna et al. (2007)	[38]	Pet (Domestic) dogs	Czech Republic	High	540	9	Low Risk
Dubna et al. (2007)	[38]	Pet (Domestic) dogs	Czech Republic	High	3780	34	Low Risk
Szabova et al. (2007)	[39]	Pet (Domestic) dogs	Slovakia	High	222	9	Low Risk
Szabova et al. (2007)	[39]	Pet (Domestic) dogs	Slovakia	High	208	12	Low Risk
Szabova et al. (2007)	[39]	Stray (wild) dogs	Slovakia	High	297	24	Low Risk
Martinez-Moreno et al. (2007)	[40]	Indeterminate (not specified) type	Spain	High	1800	269	Low Risk
Claerebout et al. (2009)	[41]	Pet (Domestic) dogs	Belgium	High	451	1	Low Risk
Claerebout et al. (2009)	[41]	Pet (Domestic) dogs	Belgium	High	351	3	Low Risk
Claerebout et al. (2009)	[41]	Pet (Domestic) dogs	Belgium	High	357	9	Low Risk
Gracenea et al. (2009)	[42]	Pet (Domestic) dogs	Spain	High	505	18	Low Risk
Tylkowska et al. (2010)	[43]	Indeterminate (not specified) type	Poland	High	763	22	Low Risk
Xhaxhiu et al. (2011)	[44]	Stray (wild) dogs	Albania	Upper middle	111	1	Moderate Risk
Balkaya and Avcıoğlu, (2011)	[45]	Pet (Domestic) dogs	Germany	High	24677	137	Low Risk
Ferreira et al. (2011)	[46]	Pet (Domestic) dogs	Portugal	High	49	0	Moderate Risk
Ferreira et al. (2011)	[46]	Pet (Domestic) dogs	Portugal	High	77	1	Moderate Risk
Balkaya et al. (2011)	[47]	Stray (wild) dogs	Turkey	Upper middle	172	66	Low Risk
Becker et al. (2012)	[48]	Stray (wild) dogs	Germany	High	445	3	Low Risk
Al-Sabi et al. (2013)	[49]	Working (domestic) dogs	Denmark	High	178	1	Low Risk
Riggio et al. (2013)	[50]	Pet (Domestic) dogs	Italy	High	239	4	Low Risk
Zanzani et al. (2014)	[51]	Pet (Domestic) dogs	Italy	High	253	0	Low Risk
Zanzani et al. (2014)	[52]	Pet (Domestic) dogs	Italy	High	463	3	Low Risk
Neves et al. (2014)	[53]	Pet (Domestic) dogs	Portugal	High	368	2	Low Risk
Papajova et al. (2014)	[54]	Indeterminate (not specified) type	Slovakia	High	578	18	Low Risk
Ortuno et al. (2014)	[55]	Pet (Domestic) dogs	Spain	High	81	2	Moderate Risk
Ortuno et al. (2014)	[55]	Working (domestic) dogs	Spain	High	88	2	Moderate Risk
Shukullari et al. (2015)	[56]	Pet (Domestic) dogs	Albania	Upper middle	602	5	Low Risk
Radev et al. (2016)	[57]	Stray (wild) dogs	Bulgaria	Upper middle	80	1	Moderate Risk
Wright et al. (2016)	[58]	Pet (Domestic) dogs	England	High	171	0	Low Risk
Guardone et al. (2016)	[59]	Pet (Domestic) dogs	Italy	High	106	0	Moderate Risk
Guardone et al. (2016)	[59]	Working (domestic) dogs	Italy	High	344	8	Low Risk
Moskvina and Ermolenko (2016)	[60]	Pet (Domestic) dogs	Russia	Upper middle	8140	970	Low Risk
Kostopoulou et al. (2017)	[61]	Working (domestic) dogs	Greece	High	72	2	Low Risk
Kostopoulou et al. (2017)	[61]	Pet (Domestic) dogs	Greece	High	529	5	Low Risk
Kostopoulou et al. (2017)	[61]	Pet (Domestic) dogs	Greece	High	278	17	Low Risk
Symeonidou et al. (2017)	[62]	Indeterminate (not specified) type	Greece	High	1036	46	Low Risk
Tamponi et al. (2017)	[63]	Pet (Domestic) dogs	Italy	High	619	7	Low Risk
Szwabe and Blaszkowska (2017)	[64]	Stray (wild) dogs	Poland	High	95	1	Moderate Risk
Studzinska et al. (2017)	[65]	Pet (Domestic) dogs	Poland	High	100	3	Moderate Risk
Mircean et al. (2017)	[66]	Working (Domestic) dogs	Romania	Upper middle	602	3	Low Risk
Mircean et al. (2017)	[66]	Pet (Domestic) dogs	Romania	Upper middle	712	11	Low Risk
Sommer et al. (2017)	[67]	Pet (Domestic) dogs	Serbia	Upper middle	134	13	Low Risk
Scaramozzino et al. (2018)	[68]	Pet (Domestic) dogs	Italy	High	2775	11	Low Risk
**North America**							
Nolan and Smith (1995)	[69]	Pet (Domestic) dogs	USA	High	8077	47	Low Risk
Coggins et al. (1998)	[70]	Stray (wild) dogs	USA	High	309	13	Low Risk
Gates et al. (2009)	[71]	Pet (Domestic) dogs	USA	High	6555	13	Low Risk
Casey Gaunt et al. (2011)	[72]	Indeterminate (not specified) type	Canada	High	457	3	Low Risk
Joffe et al. (2011)	[73]	Pet (Domestic) dogs	Canada	High	477	5	Low Risk
Joffe et al. (2011)	[73]	Pet (Domestic) dogs	Canada	High	142	8	Low Risk
Canto et al. (2011)	[73]	Stray (wild) dogs	Mexico	Upper middle	378	9	Low Risk
Schurer et al. (2012)	[74]	Pet (Domestic) dogs	Canada	High	254	40	Low Risk

Schurer et al. (2014)	[75]	Indeterminate (not specified) type	Canada	High	231	5	Low Risk
Gates and Nolan, (2014)	[76]	Pet (Domestic) dogs	USA	High	12914	28	Low Risk
Villeneuve et al. (2015)	[77]	Working (domestic) dogs	Canada	High	1086	33	Low Risk
**South America**							
Campos et al. (2016)	[78]	Pet (Domestic) dogs	Brazil	Upper middle	345	2	Low Risk
**South-East Asia**							
Kachawha and Tanwar, (2007)	[79]	Stray (wild) dogs	India	Lower middle	642	14	Low Risk
Qadir et al. (2012)	[80]	Indeterminate (not specified) type	India	Lower middle	325	3	Low Risk
Perera et al. (2013)	[81]	Pet (Domestic) dogs	Sri Lanka	Lower middle	30	0	Moderate Risk
Perera et al. (2013)	[81]	Stray (wild) dogs	Sri Lanka	Lower middle	60	11	Moderate Risk
Traub et al. (2014)	[82]	Stray (wild) dogs	India	Lower middle	411	13	Low Risk
Sudan et al. (2015)	[83]	Stray (wild) dogs	India	Lower middle	108	28	Low Risk
**Western Pacific**							
Johnston and Gasser (1993)	[84]	Pet (Domestic) dogs	Australia	High	303	0	Low Risk
Johnston and Gasser (1993)	[84]	Stray (wild) dogs	Australia	High	190	1	Low Risk
Wang et al. (2006)	[85]	Working (domestic) dogs	China	Upper middle	178	2	Low Risk
Palmer et al. (2008)	[86]	Stray (wild) dogs	Australia	High	590	0	Low Risk
Palmer et al. (2008)	[86]	Pet (Domestic) dogs	Australia	High	810	1	Low Risk
Dai et al. (2009)	[87]	Working (domestic) dogs	China	Upper middle	438	141	Low Risk
Itoh et al. (2011)	[88]	Pet (Domestic) dogs	Japan	High	109	0	Low Risk
Itoh et al. (2011)	[88]	Pet (Domestic) dogs	Japan	High	363	0	Low Risk
Itoh et al. (2011)	[88]	Pet (Domestic) dogs	Japan	High	185	0	Low Risk
Itoh et al. (2011)	[88]	Pet (Domestic) dogs	Japan	High	1169	1	Low Risk
Itoh et al. (2011)	[88]	Pet (Domestic) dogs	Japan	High	244	2	Low Risk
Itoh et al. (2011)	[88]	Pet (Domestic) dogs	Japan	High	295	2	Low Risk
Kimura et al. (2013)	[89]	Stray (wild) dogs	Japan	High	212	1	Low Risk
Ngui et al. (2014)	[90]	Working (domestic) dogs	Malaysia	Upper middle	77	4	Moderate Risk
Itoh et al. (2015)	[91]	Pet (Domestic) dogs	Japan	High	573	5	Low Risk

Table S2. Main characteristics of all eligible studies reporting prevalence of *Toxascaris leonina* infection in cats.

WHO-region/reference	Ref	Type of cats (pet or stray)	Country	Income level	Sample size	Number of test-positive samples for *Ta. leonina*	Risk of bias (quality assessment)
**Africa**							
Sowemimo et al. (2012)	[92]	Pet	Nigeria	Lower middle	200	47	Low risk
Okoye et al. (2014)	[93]	Pet	Nigeria	Lower middle	51	24	Moderate risk
Okoye et al. (2014)	[93]	Stray	Nigeria	Lower middle	68	33	Moderate risk
**Eastern Mediterranean**							
Zibaei et al. (2007)	[94]	Stray	Iran	Upper middle	114	4	Low risk
Al-khushali et al. (2007)	[95]	Stray	Iraq	Upper middle	126	41	Low risk
Schuster et al. (2009)	[96]	Stray	United Arab Emirates	High	240	2	Low risk
Abu-Madi et al. (2010)	[97]	Stray	Qatar	High	658	1	Low risk
Khalafalla et al. (2011)	[98]	Stray	Egypt	Lower middle	113	6	Low risk
Borji et al. (2011)	[99]	Stray	Iran	Upper middle	52	4	Moderate risk
Al-Rubaie et al. (2015)	[100]	Stray	Iraq	Upper middle	254	47	Low risk
Hajipour et al. (2016)	[101]	Stray	Iran	Upper middle	50	15	Moderate risk
El-Seify et al. (2017)	[102]	Stray	Egypt	Lower middle	170	14	Low risk
Hajipour (2019)	[103]	Stray	Iran	Upper middle	100	21	Moderate risk
**Europe**							
Vanparijs et al. (1991)	[26]	Stray	Belgium	High	30	0	Moderate risk
O'lorcain et al. (1994)	[104]	Pet	Czech Republic	High	135	1	Low risk
Yamaguchi et al. (1996)	[105]	Stray	England	High	11	9	Moderate risk
Overgaauw et al. (1997)	[106]	Pet	Netherlands	High	236	0	Low risk
Overgaauw et al. (1997)	[106]	Stray	Netherlands	High	56	3	Low risk
Overgaauw et al. (1997)	[106]	Pet	Netherlands	High	56	3	Low risk
Barutzki et al. (2003)	[33]	Pet	Germany	High	8560	8	Low risk
Miro et al. (2004)	[107]	Stray	Spain	High	220	1	Low risk
Miro et al. (2004)	[107]	Stray	Spain	High	365	4	Low risk
Michalczyk et al. (2008)	[108]	Pet	Poland	High	22	3	Moderate risk
Karatepe et al. (2008)	[109]	Stray	Turkey	Upper middle	72	15	Moderate risk
Näreaho et al. (2012)	[110]	Pet	Finland	High	411	1	Low risk
Capári et al. (2013)	[111]	Pet	Hungary	High	235	17	Low risk
Riggio et al. (2013)	[50]	Pet	Italy	High	81	0	Low risk
Lefkaditis et al. (2014)	[112]	Stray	Greece	High	215	2	Low risk
Zanzani et al. (2014)	[51]	Pet	Italy	High	156	12	Low risk
Öge et al. (2014)	[113]	Pet	Turkey	Upper middle	100	2	Low risk
Pallant et al. (2015)	[114]	Pet	Germany	High	60	0	Moderate risk
Moskvina et al. (2015)	[115]	Pet	Russia	Upper middle	51	4	Moderate risk
Wright et al. (2016)	[58]	Pet	England	High	131	1	Low risk
Nijsse et al. (2016)	[116]	Indeterminate (not specified) type	Netherlands	High	670	0	Low risk
Raue et al. (2017)	[117]	Pet	Germany	High	903	35	Low risk
Diakou et al. (2017)	[118]	Stray	Greece	High	150	12	Low risk
Kostopoulou et al. (2017)	[61]	Pet	Greece	High	59	0	Low risk
Kostopoulou et al. (2017)	[61]	Pet	Greece	High	205	0	Low risk
Szwabe et al. (2017)	[64]	Stray	Poland	High	68	0	Moderate risk
Symeonidou et al. (2018)	[119]	Indeterminate (not specified) type	Greece	High	1150	2	Low risk
Blasco et al. (2017)	[120]	Pet	Spain	High	423	10	Low risk
Shamaev et al. (2018)	[121]	Indeterminate (not specified) type	Russia	Upper middle	148	5	Low risk
Moskvina et al. (2018)	[122]	Pet	Russia	Upper middle	135	5	Low risk
**South America**							
Labarthe et al. (2004)	[123]	Pet	Brazil	Upper middle	36	4	Moderate risk
Labarthe et al. (2004)	[123]	Stray	Brazil	Upper middle	99	12	Moderate risk
Sommerfelt et al. (2006)	[124]	Stray	Argentina	High	465	41	Low risk
Lorenzini et al. (2007)	[125]	Pet	Brazil	Upper middle	288	1	Low risk
Campos et al. (2016)	[78]	Pet	Brazil	Upper middle	160	0	Low risk
**North America**							
Gates et al. (2009)	[71]	Pet	U.S.A	High	1566	2	Low risk
Joffe et al. (2011)	[73]	Pet	Canada	High	85	0	Low risk
Joffe et al. (2011)	[73]	Pet	Canada	High	68	0	Low risk
Lucio-Forster et al. (2011)	[126]	Pet	U.S.A	High	1322	0	Low risk
Hoopes et al. (2013)	[127]	Pet	Canada	High	635	1	Low risk
Canto et al. (2013)	[128]	Pet	Mexico	Upper middle	85	0	Moderate risk
Canto et al. (2013)	[128]	Stray	Mexico	Upper middle	273	1	Low risk
Hoopes et al. (2015)	[129]	Stray	Canada	High	161	1	Low risk
Hoopes et al. (2015)	[129]	Indeterminate (not specified) type	Canada	High	27	1	Low risk
**Western Pacific**							
Lin et al. (1990)	[130]	Pet	Taiwan	High	96	1	Low risk
McGlade et al. (2003)	[131]	Pet	Australia	High	418	9	Low risk
Palmer et al. (2008)	[86]	Sray	Australia	High	491	1	Low risk
Palmer et al. (2008)	[86]	Pet	Australia	High	572	10	Low risk
Bissett et al. (2009)	[132]	Pet	Australia	High	134	2	Low risk
Itoh et al. (2012)	[133]	Pet	Japan	High	942	2	Low risk
Beugnet et al. (2014)	[134]	Pet	Australia	High	92	5	Moderate risk
Fang et al. (2015)	[135]	Stray	China	Upper middle	39	3	Moderate risk

Table S3. Questions from the Joanna Briggs Institute Prevalence Critical Appraisal Tool.

No.	Criteria	Yes	No	Unclear	Not applicable
1	Was the sample representative of the target population?				
2	Were study participants recruited in an appropriate way?				
3	Was the sample adequate?				
4	Were the study subjects and the setting described in detail?				
5	Was the data analysis conducted with sufficient coverage of the identified sample?				
6	Were objective, standard criteria used for the measurement of the condition?				
7	Was the condition measured reliably?				
8	Was there appropriate statistical analysis?				
9	Are all important confounding factors/subgroups/differences identified and accounted for?				
10	Were subpopulations identified using objective criteria?				

Table adapted from Munn et al. (2014).

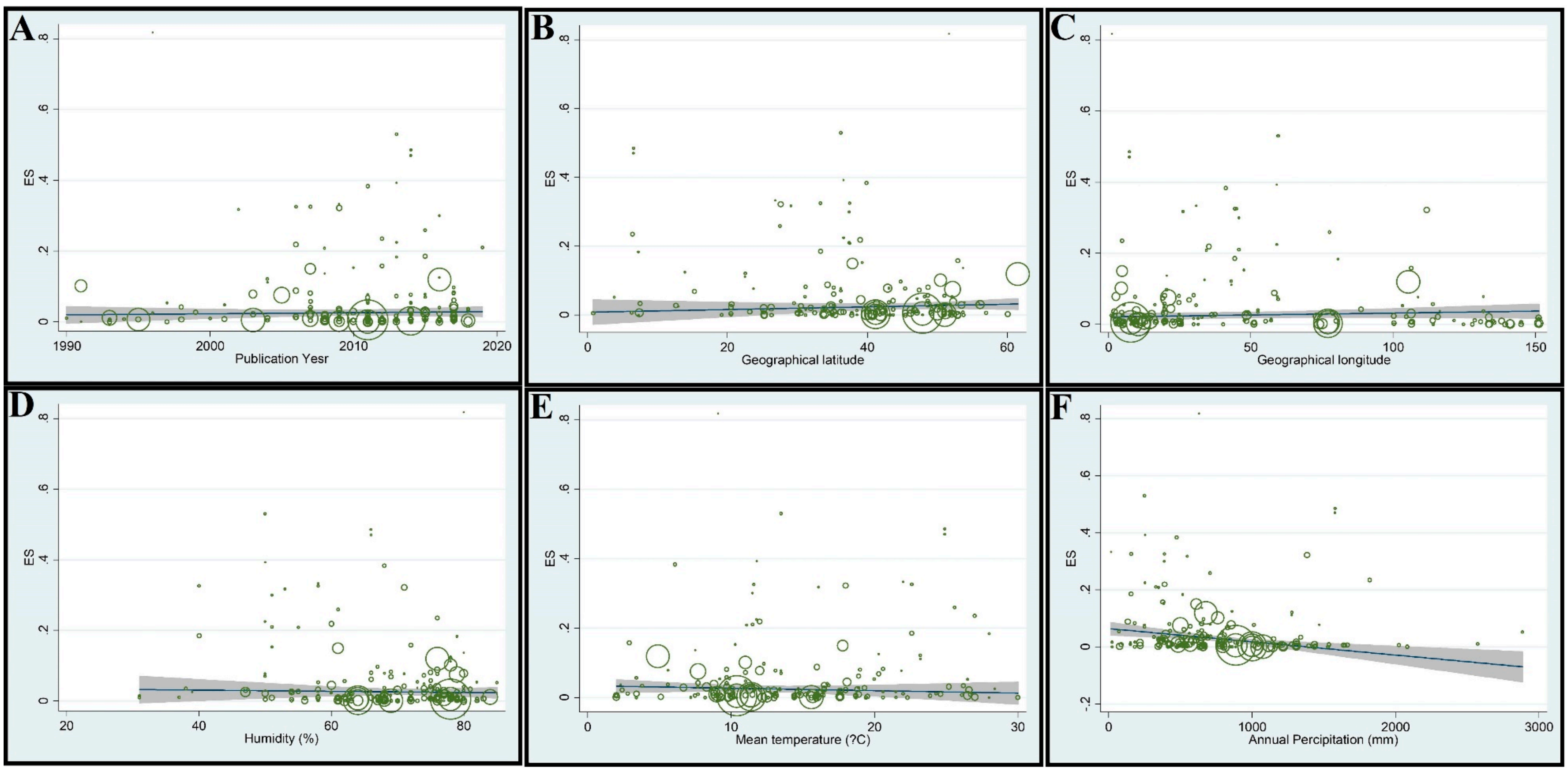

Figure 1. Results of meta-regression analyses of the prevalence of *Toxascaris leonina* infection in dogs and cats according to: (panel A) demonstrating a statistically non-significant decreasing trend in prevalence over time in more recent years; (panels B and C) geographical latitude and longitude, showing statistically non-significant downward trend in prevalence with increasing geographical latitude and longitude; (panel D) relative humidity, showing statistically significant downward trend in prevalence with increasing relative humidity; and (panels E and F) mean environmental temperature and annual precipitation, showing non-statistically significant upward and downward trend in prevalence in areas with a higher mean temperature and relative humidity, respectively.
"**ES**" refers to effect size (= prevalence rates).

References

1. Minnaar, W.; Krecek, R.; Fourie, L. Helminths in dogs from a peri-urban resource-limited community in Free State Province, South Africa. *Vet. Parasitol.* **2002**, *107*, 343–349.
2. Davoust, B.; Normand, T.; Bourry, O.; Dang, H.; Leroy, E.; Bourdoiseau, G. Epidemiological survey on gastro-intestinal and blood-borne helminths of dogs in north-east Gabon. *Onderstepoort J. Vet. Res.* **2008**, *75*, 359–364.
3. Sowemimo, O.A. Prevalence and intensity of *Toxocara canis* (Werner, 1782) in dogs and its potential public health significance in Ile-Ife, Nigeria. *J. Helminthol.* **2007**, *81*, 433–438.
4. Sowemimo, O.; Asaolu, S. Epidemiology of intestinal helminth parasites of dogs in Ibadan, Nigeria. *J. Helminthol.* **2008**, *82*, 89–93.
5. Mukaratirwa, S.; Singh, V. Prevalence of gastrointestinal parasites of stray dogs impounded by the Society for the Prevention of Cruelty to Animals (SPCA), Durban and Coast, South Africa. *J. S. Afr. Vet. Assoc.* . **2010**, *81*, 123–125.
6. Awoke, E.; Bogale, B.; Chanie, M. Intestinal nematode parasites of dogs: Prevalence and associated risk factors. *Int. J. Anim. Vet. Adv.* **2011**, *3*, 374–378.
7. Bwalya, E.C.; Nalubamba, K.S.; Hankanga, C.; Namangala, B. Prevalence of canine gastrointestinal helminths in urban Lusaka and rural Katete Districts of Zambia. *Prev. Vet. Med.* **2011**, *100*, 252–255.
8. Mahmuda, A.; Magaji, A.A.; Yakubu, Y.; Salihu, M.; Lawal, M.; Mahmud, U.; Suleiman, N.; Danmaigoro, A. Prevalence of intestinal parasites of dogs slaughtered at Mami market area, Sokoto, Nigeria. *Sci. J. Anim. Sci.* **2012**, *1*, 126–130.
9. Edosomwan, E.; Chinweuba, C. A survey on helminth parasites of dogs in Benin city, Edo State, Nigeria. *J. Vet. Med. Anim. Health* **2012**, *4*, 56–60.
10. Alvåsen, K.; Johansson, S.M.; Höglund, J.; Ssuna, R.; Emanuelson, U. A field survey on parasites and antibodies against selected pathogens in owned dogs in Lilongwe, Malawi. *J. S. Afr. Vet. Assoc.* **2016**, *87*, 1–6.
11. El-Shehabi, F.S.; Abdel-Hafez, S.K.; Kamhawi, S.A. Prevalence of intestinal helminths of dogs and foxes from Jordan. *Parasitol. Res.* **1999**, *85*, 928–934.
12. Dalimi, A.; Sattari, A.; Motamedi, G. A study on intestinal helminthes of dogs, foxes and jackals in the western part of Iran. *Vet. Parasitol.* **2006**, *142*, 129–133.
13. Sabry, M.A.; Lotfy, H.S. Captive dogs as reservoirs of some zoonotic parasites. *Res. J. Parasitol.* **2009**, *4*, 115–122.
14. Zare-Bidaki, M.; Mobedi, I.; Ahari, S.S.; Habibizadeh, S.; Naddaf, S.; Siavashi, M. Prevalence of zoonotic intestinal helminths of canids in Moghan plain, Northwestern Iran. *Iran. J. Parasitol.* **2010**, *5*, 42–51.
15. Mirzaei, M.; Fooladi, M. Prevalence of intestinal helminthes in owned dogs in Kerman city, Iran. *Asian Pac. J. Trop. Med.* **2012**, *5*, 735–737.
16. Mirzaei, M.; Fooladi, M. Coproscopy survey of gastrointestinal parasites in owned dogs of Kerman city, Iran. *Vet. Ital.* **2013**, *49*, 309–313.
17. Beiromvand, M.; Akhlaghi, L.; Massom, S.H.F.; Meamar, A.R.; Motevalian, A.; Oormazdi, H.; Razmjou, E. Prevalence of zoonotic intestinal parasites in domestic and stray dogs in a rural area of Iran. *Prev. Vet. Med.* **2013**, *109*, 162–167.
18. Adinezadeh, A.; Eshrat Beigom, K.; Mohebali, M.; Shojaee, S.; Rokni, M.B.; Zarei, Z.; Mowlavi, G. Endoparasites of stray dogs in Mashhad, Khorasan Razavi province, Northeast Iran with special reference to zoonotic parasites. *Iran. J. Parasitol.* **2013**, *8*, 459–466.
19. Ahmed, W.; Mousa, W.; Aboelhadid, S.; Tawfik, M. Prevalence of zoonotic and other gastrointestinal parasites in police and house dogs in Alexandria, Egypt. *Vet. World.* **2014**, *7*, 275–280.
20. Awadallah, M.A.; Salem, L.M. Zoonotic enteric parasites transmitted from dogs in Egypt with special concern to *Toxocara canis* infection. *Vet. world* **2015**, *8*, 946–957.
21. Sardarian, K.; Maghsood, A.; Ghiasian, S.; Zahirnia, A. Prevalence of zoonotic intestinal parasites in household and stray dogs in rural areas of Hamadan, Western Iran. *Trop Biomed.* **2015**, *32*, 240–246.
22. Emamapour, S.R.; Borji, H.; Nagibi, A. An epidemiological survey on intestinal helminths of stray dogs in Mashhad, North-east of Iran. *J. Parasit. Dis.* **2015**, *39*, 266–271.
23. Sarvi, S.; Daryani, A.; Sharif, M.; Rahimi, M.T.; Azami, D.; Marhaba, Z.; Ahmadpour, E.; Mizani, A. Domestic dog as a human health hazard in north of Iran. *J. Parasit. Dis.* **2016**, *40*, 930–934.
24. Kohansal, M.H.; Fazaeli, A.; Nourian, A.; Haniloo, A.; Kamali, K. Dogs' gastrointestinal parasites and their association with public health in Iran. *J. Vet. Res.* **2017**, *61*, 189–195.
25. Al-Jassim, K.B.; Mahmmod, Y.S.; Salem, Z.M.; Al-Jubury, A. Epidemiological investigation of gastrointestinal parasites in dog populations in Basra province, Southern Iraq. *J. Parasit. Dis.* **2017**, *41*, 1006–1013.
26. Vanparijs, O.; Hermans, L.; Van Der Flaes, L. Helminth and protozoan parasites in dogs and cats in Belgium. *Vet. Parasitol.* **1991**, *38*, 67–73.
27. Epe, C.; Ising-Volmer, S.; Stoye, M. Parasitological fecal studies of equids, dogs, cats and hedgehogs during the years 1984-1991. *Dtsch. Tierarztl. Wochenschr.* **1993**, *100*, 426–428.
28. Svobodova, V.; Konvalinova, J.; Svoboda, M. Coprological and serological findings in dogs and cats with giardiosis and cryptosporidiosis. *Acta Vet. Brno.* **1995**, *64*, 257–262.
29. Causape, A.; Quilez, J.; Sanchez-Acedo, C.; Del Cacho, E. Prevalence of intestinal parasites, including *Cryptosporidium parvum*, in dogs in Zaragoza city, Spain. *Vet. Parasitol.* **1996**, *67*, 161–167.
30. Overgaauw, P.; Boersema, J. Nematode infections in dog breeding kennels in The Netherlands, with special reference to *Toxocara*. *Vet. Quart.* **1998**, *20*, 12–15.
31. Maśnik, E. Relationships between the prevalence of *Toxocara* eggs in dogs' faeces and soil. *Wiad. Parazytol.* **2000**, *46*, 239–244.
32. Fok, E.; Szatmari, V.; Busak, K.; Rozgonyi, F. Epidemiology: Prevalence of intestinal parasites in dogs in some urban and rural areas of Hungary. *Vet. Quart.* **2001**, *23*, 96–98.
33. Barutzki, D.; Schaper, R. Endoparasites in dogs and cats in Germany 1999–2002. *Parasitol. Res.* **2003**, *90*, S148-S150.
34. Benito, A.; Carmena, D.; Postigo, I.; Estibalez, J.; Martinez, J.; Guisantes, J. Intestinal helminths in dogs in Alava, North of Spain. *Res. Rev. Parasitol.* **2003**, *63*, 121–126.
35. Borecka, A. Prevalence of intestinal nematodes of dogs in the Warsaw area, Poland. *Helminthologia* **2005**, *42*, 35–39.
36. Sager, H.; Moret, C.S.; Grimm, F.; Deplazes, P.; Doherr, M.; Gottstein, B. Coprological study on intestinal helminths in Swiss dogs: temporal aspects of anthelminthic treatment. *Parasitol. Res.* **2006**, *98*, 333–338.
37. Senlik, B.; Cirak, V.; Karabacak, A. Intestinal nematode infections in Turkish military dogs with special reference to *Toxocara canis*. *J. Helminthol.* **2006**, *80*, 299–303.
38. Dubná, S.; Langrová, I.; Nápravník, J.; Jankovská, I.; Vadlejch, J.; Pekár, S.; Fechtner, J. The prevalence of intestinal parasites in dogs from Prague, rural areas, and shelters of the Czech Republic. *Vet. Parasitol.* **2007**, *145*, 120–128.
39. Szabová, E.; Juriš, P.; Miterpáková, M.; Antolová, D.; Papajová, I.; Šefčíková, H. Prevalence of important zoonotic parasites in dog populations from the Slovak Republic. *Helminthologia* **2007**, *44*, 170–176.
40. Martinez-Moreno, F.; Hernández, S.; López-Cobos, E.; Becerra, C.; Acosta, I.; Martínez-Moreno, A. Estimation of canine intestinal parasites in Cordoba (Spain) and their risk to public health. *Vet. Parasitol.* **2007**, *143*, 7–13.
41. Claerebout, E.; Casaert, S.; Dalemans, A.-C.; De Wilde, N.; Levecke, B.; Vercruysse, J.; Geurden, T. *Giardia* and other intestinal parasites in different dog populations in Northern Belgium. *Vet. Parasitol.* **2009**, *161*, 41–46.
42. Gracenea, M.; Gómez, M.S.; Torres, J. Prevalence of intestinal parasites in shelter dogs and cats in the metropolitan area of Barcelona (Spain). *Acta Parasitol.* **2009**, *54*, 73–77.
43. Tylkowska, A.; Pilarczyk, B.; Gregorczyk, A.; Templin, E. Gastrointestinal helminths of dogs in Western Pomerania, Poland. *Wiad. Parazytol.* **2010**, *56*, 269–276.
44. Xhaxhiu, D.; Kusi, I.; Rapti, D.; Kondi, E.; Postoli, R.; Rinaldi, L.; Dimitrova, Z.M.; Visser, M.; Knaus, M.; Rehbein, S. Principal intestinal parasites of dogs in Tirana, Albania. *Parasitol. Res.* **2011**, *108*, 341–353.
45. Barutzki, D.; Schaper, R. Results of parasitological examinations of faecal samples from cats and dogs in Germany between 2003 and 2010. *Parasitol. Res.* **2011**, *109*, 45–60.
46. Ferreira, F.; Pereira-Baltasar, P.; Parreira, R.; Padre, L.; Vilhena, M.; Tavira, L.T.; Atouguia, J.; Centeno-Lima, S. Intestinal parasites in dogs and cats from the district of Évora, Portugal. *Vet. Parasitol.* **2011**, *179*, 242–245.

47. Balkaya, İ.; Avcıoğlu, H. Gastro-intestinal helminths detected by coprological examination in stray dogs in the Erzurum province Turkey. *Kafkas Univ. Vet. Fak. Derg.* **2011**, *17*, 43–46.
48. Becker, A.-C.; Rohen, M.; Epe, C.; Schnieder, T. Prevalence of endoparasites in stray and fostered dogs and cats in Northern Germany. *Parasitol. Res.* **2012**, *111*, 849–857.
49. Al-Sabi, M.N.; Kapel, C.M.; Johansson, A.; Espersen, M.C.; Koch, J.; Willesen, J.L. A coprological investigation of gastrointestinal and cardiopulmonary parasites in hunting dogs in Denmark. *Vet. Parasitol.* **2013**, *196*, 366–372.
50. Riggio, F.; Mannella, R.; Ariti, G.; Perrucci, S. Intestinal and lung parasites in owned dogs and cats from central Italy. *Vet. Parasitol.* **2013**, *193*, 78–84.
51. Zanzani, S.A.; Gazzonis, A.L.; Scarpa, P.; Berrilli, F.; Manfredi, M.T. Intestinal parasites of owned dogs and cats from metropolitan and micropolitan areas: prevalence, zoonotic risks, and pet owner awareness in northern Italy. *BioMed Res. Int.* **2014**, *2014*, 696508.
52. Zanzani, S.A.; Di Cerbo, A.R.; Gazzonis, A.L.; Genchi, M.; Rinaldi, L.; Musella, V.; Cringoli, G.; Manfredi, M.T. Canine fecal contamination in a metropolitan area (Milan, north-western Italy): prevalence of intestinal parasites and evaluation of health risks. *Sci. World J.* **2014**, *2014*, 696508.
53. Neves, D.; Lobo, L.; Simões, P.B.; Cardoso, L. Frequency of intestinal parasites in pet dogs from an urban area (Greater Oporto, northern Portugal). *Vet. Parasitol.* **2014**, *200*, 295–298.
54. Papajová, I.; Pipiková, J.; Papaj, J.; Čižmár, A. Parasitic contamination of urban and rural environments in the Slovak Republic: dog's excrements as a source. *Helminthologia* **2014**, *51*, 273–280.
55. Ortuño, A.; Scorza, V.; Castellà, J.; Lappin, M. Prevalence of intestinal parasites in shelter and hunting dogs in Catalonia, Northeastern Spain. *Vet. J.* **2014**, *199*, 465–467.
56. Shukullari, E.; Hamel, D.; Rapti, D.; Pfister, K.; Visser, M.; Winter, R.; Rehbein, S. Parasites and vector-borne diseases in client-owned dogs in Albania. Intestinal and pulmonary endoparasite infections. *Parasitol. Res.* **2015**, *114*, 4579–4590.
57. Radev, V.; Lalkovski, N.; Zhelyazkov, P.; Kostova, T.; Sabev, P.; Nedelchev, N.; Vassileva, R. Prevalence of gastrointestinal parasites and *Dirofilaria* spp. in stray dogs from some regions in Bulgaria. *Bulg. J. Vet. Med.* **2016**, *19*, 57–62.
58. Wright, I.; Stafford, K.; Coles, G. The prevalence of intestinal nematodes in cats and dogs from Lancashire, north-west England. *J. Small Anim. Pract.* **2016**, *57*, 393–395.
59. Guardone, L.; Magi, M.; Prati, M.; Macchioni, F. Cardiorespiratory and gastrointestinal parasites of dogs in north-west Italy. *Helminthologia* **2016**, *53*, 318–325.
60. Moskvina, T.; Ermolenko, A. Helminth infections in domestic dogs from Russia. *Vet. World* **2016**, *9*, 1248.
61. Kostopoulou, D.; Claerebout, E.; Arvanitis, D.; Ligda, P.; Voutzourakis, N.; Casaert, S.; Sotiraki, S. Abundance, zoonotic potential and risk factors of intestinal parasitism amongst dog and cat populations: The scenario of Crete, Greece. *Parasite. Vectors* **2017**, *10*, 43.
62. Symeonidou, I.; Gelasakis, A.; Arsenopoulos, K.; Schaper, R.; Papadopoulos, E. Regression models to assess the risk factors of canine gastrointestinal parasitism. *Vet. Parasitol.* **2017**, *248*, 54–61.
63. Tamponi, C.; Varcasia, A.; Pinna, S.; Melis, E.; Melosu, V.; Zidda, A.; Sanna, G.; Pipia, A.; Zedda, M.; Pau, S. Endoparasites detected in faecal samples from dogs and cats referred for routine clinical visit in Sardinia, Italy. *Vet. Parasitol. Reg. Stud. Reports* **2017**, *10*, 13–17.
64. Szwabe, K.; Blaszkowska, J. Stray dogs and cats as potential sources of soil contamination with zoonotic parasites. *Ann. Agric. Environ. Med.* **2017**, *24*, 39–43.
65. Studzińska, M.B.; Demkowska-Kutrzepa, M.; Borecka, A.; Meisner, M.; Tomczuk, K.; Roczeń-Karczmarz, M.; Kłapeć, T.; Abbass, Z.; Cholewa, A. Variations in the rate of infestations of dogs with zoonotic nematodes and the contamination of soil in different environments. *Int. J. Environ. Res. Public Health* **2017**, *14*, 1003.
66. Mircean, V.; Dumitrache, M.O.; Mircean, M.; Colosi, H.A.; Györke, A. Prevalence and risk factors associated with endoparasitic infection in dogs from Transylvania (Romania): a retrospective study. *Vet. Parasitol.* **2017**, *243*, 157–161.
67. Sommer, M.F.; Zdravković, N.; Vasić, A.; Grimm, F.; Silaghi, C. Gastrointestinal parasites in shelter dogs from Belgrade, Serbia. *Vet. Parasitol. Reg. Stud. Reports* **2017**, *7*, 54–57.
68. Scaramozzino, P.; Carvelli, A.; Iacoponi, F.; De Liberato, C. Endoparasites in household and shelter dogs from central Italy. *Int J Vet Sci Med.* **2018**, *6*, 45–47.
69. Nolan, T.J.; Smith, G. Time series analysis of the prevalence of endoparasitic infections in cats and dogs presented to a veterinary teaching hospital. *Vet. Parasitol.* **1995**, *59*, 87–96.
70. Coggins, J.R. Effect of season, sex, and age on prevalence of parasitism in dogs from southeastern Wisconsin. *J. Helminthol. Soc. Wash.* **1998**, *65*, 219–226.
71. Gates, M.C.; Nolan, T.J. Endoparasite prevalence and recurrence across different age groups of dogs and cats. *Vet. Parasitol.* **2009**, *166*, 153–158.
72. Gaunt, C.M.; Carr, A.P. A survey of intestinal parasites in dogs from Saskatoon, Saskatchewan. *Can. Vet. J.* **2011**, *52*, 497–500.
73. Joffe, D.; Van Niekerk, D.; Gagné, F.; Gilleard, J.; Kutz, S.; Lobingier, R. The prevalence of intestinal parasites in dogs and cats in Calgary, Alberta. *Can. Vet. J.* **2011**, *52*, 1323–1328.
74. Schurer, J.M.; Hill, J.E.; Fernando, C.; Jenkins, E.J. Sentinel surveillance for zoonotic parasites in companion animals in indigenous communities of Saskatchewan. *Am. J. Trop. Med. Hyg.* **2012**, *87*, 495–498.
75. Schurer, J.M.; Hamblin, B.; Davenport, L.; Wagner, B.; Jenkins, E.J. Rural origin, age, and endoparasite fecal prevalence in dogs surrendered to the Regina Humane Society, 2013. *Can. Vet. J.* **2014**, *55*, 1192–1195.
76. Gates, M.C.; Nolan, T.J. Declines in canine endoparasite prevalence associated with the introduction of commercial heartworm and flea preventatives from 1984 to 2007. *Vet. Parasitol.* **2014**, *204*, 265–268.
77. Villeneuve, A.; Polley, L.; Jenkins, E.; Schurer, J.; Gilleard, J.; Kutz, S.; Conboy, G.; Benoit, D.; Seewald, W.; Gagné, F. Parasite prevalence in fecal samples from shelter dogs and cats across the Canadian provinces. *Parasite. Vectors* **2015**, *8*, 281.
78. Campos, D.R.; Oliveira, L.C.; de Siqueira, D.F.; Perin, L.R.; Campos, N.C.; Aptekmann, K.P.; Martins, I.V.F. Prevalence and risk factors associated with endoparasitosis of dogs and cats in Espírito Santo, Brazil. *Acta Parasitol.* **2016**, *61*, 544–548.
79. Kachawha, S.; Tanwar, R. Prevalence of worm infestations in stray dogs in and around Jodhpur. *J. Vet. Parasitol.* **2007**, *21*, 171–172.
80. Qadir, S.; Dixit, A.K.; Dixit, P. Prevalence and intensity of canine gastrointestinal helminths in Jabalpur, Madhya Pradesh. *J. Vet. Parasitol.* **2012**, *26*, 23–26.
81. Perera, P.; Rajapakse, R.; Rajakaruna, R. Gastrointestinal parasites of dogs in Hantana area in the Kandy District. *J. Natl. Sci. Found Sri.* **2013**, *41*, 81–91.
82. Traub, R.J.; Pednekar, R.P.; Cuttell, L.; Porter, R.B.; Rani, P.A.A.M.; Gatne, M.L. The prevalence and distribution of gastrointestinal parasites of stray and refuge dogs in four locations in India. *Vet. Parasitol.* **2014**, *205*, 233–238.
83. Sudan, V.; Jaiswal, A.K.; Shanker, D.; Kanojiya, D.; Sachan, A. Prevalence of endoparasitic infections of non descript dogs in Mathura, Uttar Pradesh. *J. Parasit. Dis.* **2015**, *39*, 491–494.
84. Johnston, J.; Gasser, R. Copro-parasitological survey of dogs in southern Victoria. *Aust. Vet. Pract.* **1993**, *23*, 127–131.
85. Wang, C.; Qiu, J.; Zhao, J.P.; Xu, L.; Yu, W.; Zhu, X. Prevalence of helminthes in adult dogs in Heilongjiang Province, the People's Republic of China. *Parasitol. Res.* **2006**, *99*, 627–630.
86. Palmer, C.S.; Thompson, R.A.; Traub, R.J.; Rees, R.; Robertson, I.D. National study of the gastrointestinal parasites of dogs and cats in Australia. *Vet. Parasitol.* **2008**, *151*, 181–190.
87. Dai, R.; Li, Z.; Li, F.; Liu, D.; Liu, W.; Liu, G.; He, S.; Tan, M.; Lin, R.; Liu, Y. Severe infection of adult dogs with helminths in Hunan Province, China poses significant public health concerns. *Vet. Parasitol.* **2009**, *160*, 348–350.
88. Itoh, N.; Kanai, K.; Tominaga, H.; Kawamata, J.; Kaneshima, T.; Chikazawa, S.; Hori, Y.; Hoshi, F.; Higuchi, S. Giardia and other intestinal parasites in dogs from veterinary clinics in Japan. *Parasitol. Res.* **2011**, *109*, 253–256.
89. Kimura, A.; Morishima, Y.; Nagahama, S.; Horikoshi, T.; Edagawa, A.; Kawabuchi-Kurata, T.; Sugiyama, H.; Yamasaki, H. A coprological survey of intestinal helminthes in stray dogs captured in osaka prefecture, Japan. *J. Vet. Med. Sci.* **2013**, *75*, 1409–1411.
90. Ngui, R.; Lee, S.C.; Yap, N.J.; Tan, T.K.; Aidil, R.M.; Chua, K.H.; Aziz, S.; Sulaiman, W.Y.W.; Ahmad, A.F.; Mahmud, R. Gastrointestinal parasites in rural dogs and cats in Selangor and Pahang states in Peninsular Malaysia. *Acta Parasitol.* **2014**, *59*, 737–744.
91. Itoh, N.; Kanai, K.; Kimura, Y.; Chikazawa, S.; Hori, Y.; Hoshi, F. Prevalence of intestinal parasites in breeding kennel dogs in Japan. *Parasitol. Res.* **2015**, *114*, 1221–1224.
92. Sowemimo, O.A. Prevalence and intensity of gastrointestinal parasites of domestic cats in Ode Irele and Oyo communities, Southwest Nigeria. *J. Parasitol. Vector Biol.* **2012**, *4*, 7–13.
93. Okoye, I.C.; Obiezue, N.R.; Okoh, F.N.; Amadi, E.C. Descriptive epidemiology and seasonality of intestinal parasites of cats in Southeast Nigeria. *Comp. Clin. Path.* **2014**, *23*, 999–1005.
94. Zibaei, M.; Sadjjadi, S.M.; Sarkari, B. Prevalence of *Toxocara cati* and other intestinal helminths in stray cats in Shiraz, Iran. *Trop Biomed.* **2007**, *24*, 39–43.

95. Al-khushali, M.N. Prevalence of zoonotic parasites in stray cats in Baghdad. *Iraq. Acad. Sci. J.* **2007**, *6*, 152–156.
96. Schuster, R.K.; Thomas, K.; Sivakumar, S.; O'Donovan, D. The parasite fauna of stray domestic cats (Felis catus) in Dubai, United Arab Emirates. *Parasitol. Res.* **2009**, *105*, 125–134.
97. Abu-Madi, M.A.; Behnke, J.M.; Prabhaker, K.; Al-Ibrahim, R.; Lewis, J.W. Intestinal helminths of feral cat populations from urban and suburban districts of Qatar. *Vet. Parasitol.* **2010**, *168*, 284–292.
98. Khalafalla, R.E. A survey study on gastrointestinal parasites of stray cats in northern region of Nile delta, Egypt. *PLoS One.* **2011**, *6*, e20283.
99. Borji, H.; Razmi, G.; Ahmadi, A.; Karami, H.; Yaghfoori, S.; Abedi, V. A survey on endoparasites and ectoparasites of stray cats from Mashhad (Iran) and association with risk factors. *J. Parasit. Dis.* **2011**, *35*, 202–206.
100. Al-Rubaie, A.; Mhaisen, F.T.; Al-Tae, A. Survey of some gastrointestinal cestodes and nematodes from stray cats at Baghdad city, Iraq. *Am. J. Biol. Lif. Sci.* **2015**, *3*, 246–253.
101. Hajipour, N.; Baran, A.I.; Yakhchali, M.; Khojasteh, S.M.B.; Hesari, F.S.; Esmaeilnejad, B.; Arjmand, J. A survey study on gastrointestinal parasites of stray cats in Azarshahr,(East Azerbaijan province, Iran). *J. Parasit. Dis.* **2016**, *40*, 1255–1260.
102. El-Seify, M.A.; Aggour, M.G.; Sultan, K.; Marey, N.M. Gastrointestinal helminths of stray cats in Alexandria, Egypt: A fecal examination survey study. *Vet Parasitol Reg Stud Reports* **2017**, *8*, 104–106.
103. Hajipour, N. A survey on the prevalence of *Toxocara cati*, *Toxocara canis* and *Toxascaris leonina* eggs in stray dogs and cats' faeces in Northwest of Iran: a potential risk for human health. *Trop Biomed.* **2019**, *36*, 143–151.
104. O'lorcain, P. Epidemiology of *Toxocara* spp. in stray dogs and cats in Dublin, Ireland. *J. Helminthol.* **1994**, *68*, 331–336.
105. Yamaguchi, N.; Macdonald, D.; Passanisi, W.; Harbour, D.; Hopper, C. Parasite prevalence in free-ranging farm cats, Felis silvestris catus. *Epidemiol. Infect.* **1996**, *116*, 217–223.
106. Overgaauw, P. Prevalence of intestinal nematodes of dogs and cats in the Netherlands. *Vet. Quart.* **1997**, *19*, 14–17.
107. Miró, G.; Montoya, A.; Jiménez, S.; Frisuelos, C.; Mateo, M.; Fuentes, I. Prevalence of antibodies to *Toxoplasma gondii* and intestinal parasites in stray, farm and household cats in Spain. *Vet. Parasitol.* **2004**, *126*, 249–255.
108. Michalczyk, M.; Sokół, R. The incidence of internal parasites in dogs and cats as dependent on the level of awareness among owners. *Wiad. Parazytol.* **2008**, *54*, 245–247.
109. Karatepe, B.; Babür, C.; Karatepe, M.; Kiliç, S.; Dündar, B. Prevalence of *Toxoplasma gondii* antibodies and intestinal parasites in stray cats from Nigde, Turkey. *Ital. J. Anim. Sci.* **2008**, *7*, 113–118.
110. Näreaho, A.; Puomio, J.; Saarinen, K.; Jokelainen, P.; Juselius, T.; Sukura, A. Feline intestinal parasites in Finland: prevalence, risk factors and anthelmintic treatment practices. *J. Feline. Med. Surg.* **2012**, *14*, 378–383.
111. Capári, B.; Hamel, D.; Visser, M.; Winter, R.; Pfister, K.; Rehbein, S. Parasitic infections of domestic cats, Felis catus, in western Hungary. *Vet. Parasitol.* **2013**, *192*, 33–42.
112. Lefkaditis, M.; Paştiu, A.; Rodi-Buriel, A.; Sossidou, A.; Panorias, A.; Eleftheriadis, T.; Cozma, V.; Mihalca, A. Helminth burden in stray cats from Thessaloniki, Greece. *Helminthologia* **2014**, *51*, 73–76.
113. Öge, H.; Öge, S.; Özbakış, G.; Gürcan, S. Comparison of *Toxocara* eggs in hair and faecal samples from owned dogs and cats collected in Ankara, Turkey. *Vet. Parasitol.* **2014**, *206*, 227–231.
114. Pallant, L.; Barutzki, D.; Schaper, R.; Thompson, R.A. The epidemiology of infections with *Giardia* species and genotypes in well cared for dogs and cats in Germany. *Parasite. Vectors* **2015**, *8*, 2.
115. Moskvina, T.; Zheleznova, L. A survey on endoparasites and ectoparasites in domestic dogs and cats in Vladivostok, Russia 2014. *Vet. Parasitol. Reg. Stud. Reports* **2015**, *1*, 31–34.
116. Nijsse, R.; Ploeger, H.; Wagenaar, J.; Mughini-Gras, L. Prevalence and risk factors for patent *Toxocara* infections in cats and cat owners' attitude towards deworming. *Parasitol. Res.* **2016**, *115*, 4519–4525.
117. Raue, K.; Heuer, L.; Böhm, C.; Wolken, S.; Epe, C.; Strube, C. 10-year parasitological examination results (2003 to 2012) of faecal samples from horses, ruminants, pigs, dogs, cats, rabbits and hedgehogs. *Parasitol. Res.* **2017**, *116*, 3315–3330.
118. Diakou, A.; Di Cesare, A.; Accettura, P.M.; Barros, L.; Iorio, R.; Paoletti, B.; di Regalbono, A.F.; Halos, L.; Beugnet, F.; Traversa, D. Intestinal parasites and vector-borne pathogens in stray and free-roaming cats living in continental and insular Greece. *PLoS Negl. Trop. dis.* **2017**, *11*, e0005335.
119. Symeonidou, I.; Gelasakis, A.I.; Arsenopoulos, K.; Angelou, A.; Beugnet, F.; Papadopoulos, E. Feline gastrointestinal parasitism in Greece: emergent zoonotic species and associated risk factors. *Parasite. Vectors* **2018**, *11*, 227.
120. Blasco, X.; Salas, A.; Manuelian, C.; Torre, C.; Ortuño, A. Intestinal Parasitic Infection in Multi-Cat Shelters in Catalonia. *Israel. J. Vet. Med.* **2017**, *72*, 16–21.
121. Shamaev Nikolaj, D.; Shuralev Eduard, A.; Galiullina Alina, V.; Fedotova Anastasiya, Y.; Mukminov Malik, N. Prevalence and risk factors of *Toxoplasma*-like and intestinal parasites in cats from urbanized area of Tatarstan, Russia. *Res. J. Pharm. Biol. Chem. Sci.* **2018**, *9*, 465–471.
122. Moskvina, T.V.; Atopkin, D.M. The prevalence of intestinal parasites of domestic cats and dogs in Vladivostok, Russia during 2014–2017. *Zool. Ecol.* **2018**, *28*, 180–184.
123. Labarthe, N.; Serrão, M.L.; Ferreira, A.M.R.; Almeida, N.K.; Guerrero, J. A survey of gastrointestinal helminths in cats of the metropolitan region of Rio de Janeiro, Brazil. *Vet. Parasitol.* **2004**, *123*, 133–139.
124. Sommerfelt, I.; Cardillo, N.; López, C.; Ribicich, M.; Gallo, C.; Franco, A. Prevalence of *Toxocara cati* and other parasites in cats' faeces collected from the open spaces of public institutions: Buenos Aires, Argentina. *Vet. Parasitol.* **2006**, *140*, 296–301.
125. Lorenzini, G.; Tasca, T.; De Carli, G.A. Prevalence of intestinal parasites in dogs and cats under veterinary care in Porto Alegre, Rio Grande do Sul, Brazil. *Braz. J. Vet. Res. Anim. Sci.* **2007**, *44*, 137–145.
126. Lucio-Forster, A.; Bowman, D.D. Prevalence of fecal-borne parasites detected by centrifugal flotation in feline samples from two shelters in upstate New York. *J. Feline. Med. Surg.* **2011**, *13*, 300–303.
127. Hoopes, J.H.; Polley, L.; Wagner, B.; Jenkins, E.J. A retrospective investigation of feline gastrointestinal parasites in western Canada. *Can. Vet. J.* **2013**, *54*, 359–362.
128. Canto, G.J.; Guerrero, R.I.; Olvera-Ramírez, A.M.; Milian, F.; Mosqueda, J.; Aguilar-Tipacamu, G. Prevalence of fleas and gastrointestinal parasites in free-roaming cats in central Mexico. *PloS One.* **2013**, *8*, e60744.
129. Hoopes, J.; Hill, J.E.; Polley, L.; Fernando, C.; Wagner, B.; Schurer, J.; Jenkins, E. Enteric parasites of free-roaming, owned, and rural cats in prairie regions of Canada. *Can. Vet. J.* **2015**, *56*, 495–501.
130. Lin, D.-S.; Lai, S.-S.; Bowman, D.D.; Jacobson, R.H.; Barr, M.C.; Giovengo, S.L. Feline immunodeficiency virus, feline leukaemia virus, *Toxoplasma gondii*, and intestinal parasitic infections in Taiwanese cats. *Br. Vet. J.* **1990**, *146*, 468–475.
131. McGlade, T.; Robertson, I.; Elliot, A.; Read, C.; Thompson, R. Gastrointestinal parasites of domestic cats in Perth, Western Australia. *Vet. Parasitol.* **2003**, *117*, 251–262.
132. Bissett, S.A.; Stone, M.L.; Malik, R.; Norris, J.M.; O'Brien, C.; Mansfield, C.S.; Nicholls, J.M.; Griffin, A.; Gookin, J.L. Observed occurrence of *Tritrichomonas foetus* and other enteric parasites in Australian cattery and shelter cats. *J. Feline. Med. Surg.* **2009**, *11*, 803–807.
133. Itoh, N.; Ikegami, H.; Takagi, M.; Ito, Y.; Kanai, K.; Chikazawa, S.; Hori, Y.; Hoshi, F.; Higuchi, S. Prevalence of intestinal parasites in private-household cats in Japan. *J. Feline. Med. Surg.* **2012**, *14*, 436–439.
134. Beugnet, F.; Bourdeau, P.; Chalvet-Monfray, K.; Cozma, V.; Farkas, R.; Guillot, J.; Halos, L.; Joachim, A.; Losson, B.; Miró, G. Parasites of domestic owned cats in Europe: co-infestations and risk factors. *Parasite. Vectors* **2014**, *7*, 291.
135. Fang, F.; Li, J.; Huang, T.; Guillot, J.; Huang, W. Zoonotic helminths parasites in the digestive tract of feral dogs and cats in Guangxi, China. *BMC Vet. Res.* **2015**, *11*, 211.
